# Novel Digital Features Discriminate Between Drought Resistant and Drought Sensitive Rice Under Controlled and Field Conditions

**DOI:** 10.3389/fpls.2018.00492

**Published:** 2018-04-17

**Authors:** Lingfeng Duan, Jiwan Han, Zilong Guo, Haifu Tu, Peng Yang, Dong Zhang, Yuan Fan, Guoxing Chen, Lizhong Xiong, Mingqiu Dai, Kevin Williams, Fiona Corke, John H. Doonan, Wanneng Yang

**Affiliations:** ^1^National Key Laboratory of Crop Genetic Improvement, National Center of Plant Gene Research, Agricultural Bioinformatics Key Laboratory of Hubei Province, College of Engineering, Huazhong Agricultural University, Wuhan, China; ^2^National Plant Phenomics Centre, Institute of Biological, Environmental and Rural Sciences, Aberystwyth University, Aberystwyth, United Kingdom

**Keywords:** high-throughput phenotyping, drought response, stay-green, leaf-rolling, RGB image analysis

## Abstract

Dynamic quantification of drought response is a key issue both for variety selection and for functional genetic study of rice drought resistance. Traditional assessment of drought resistance traits, such as stay-green and leaf-rolling, has utilized manual measurements, that are often subjective, error-prone, poorly quantified and time consuming. To relieve this phenotyping bottleneck, we demonstrate a feasible, robust and non-destructive method that dynamically quantifies response to drought, under both controlled and field conditions. Firstly, RGB images of individual rice plants at different growth points were analyzed to derive 4 features that were influenced by imposition of drought. These include a feature related to the ability to stay green, which we termed greenness plant area ratio (GPAR) and 3 shape descriptors [total plant area/bounding rectangle area ratio (TBR), perimeter area ratio (PAR) and total plant area/convex hull area ratio (TCR)]. Experiments showed that these 4 features were capable of discriminating reliably between drought resistant and drought sensitive accessions, and dynamically quantifying the drought response under controlled conditions across time (at either daily or half hourly time intervals). We compared the 3 shape descriptors and concluded that PAR was more robust and sensitive to leaf-rolling than the other shape descriptors. In addition, PAR and GPAR proved to be effective in quantification of drought response in the field. Moreover, the values obtained in field experiments using the collection of rice varieties were correlated with those derived from pot-based experiments. The general applicability of the algorithms is demonstrated by their ability to probe archival *Miscanthus* data previously collected on an independent platform. In conclusion, this image-based technology is robust providing a platform-independent tool for quantifying drought response that should be of general utility for breeding and functional genomics in future.

## Introduction

Rice, a staple food that feeds half of the world's population (Yang et al., 2013), is one of the most water-hungry crops (Xia et al., 2006). Widespread pollution, climatic change, and a growing population further aggravate the water shortage problem. Thus, water scarcity has become an urgent global and environmental problem (Hussain and Mumtaz, 2014; Trenberth et al., 2014). Furthermore, water availability varies geospatially and across seasons, making drought a key restraining factor in rice production (Uga et al., 2013). When drought stress occurs during the reproductive stage, rice yield is very significantly reduced (Venuprasad et al., 2009; Vikram et al., 2011).

Drought resistant varieties assist in maintaining and improving rice yield under such conditions. Therefore, as in many crops, one of the major targets in breeding is to develop drought resistant varieties. To accelerate the identification of novel drought resistant varieties in breeding programs, a rapid species-agnostic method for evaluation of drought-resistance under diverse conditions is a necessary prerequisite. Since cost-effective solutions are likely to be based on visible attributes, we explored the readily observable (macroscopic) reactions of leaves to drought.

As with many species of grass, rice leaves roll, and change their overall geometry in response to drought conditions and then recover when water is available (Begg et al., 1980; Tardieu, 2013). Therefore, leaf rolling and general leaf geometry could be useful indicators and have already been widely used to identify drought resistant varieties (Richards et al., 2010). Variation in leaf rolling has been recorded using visual scores (O'Toole and Cruz, 1980; Turner, 1997). Color change is another commonly reported reaction, as leaves tend to become yellow when subjected to drought. Therefore, prolonged persistence of green color (sometimes also referred to as the stay-green trait) could be an appropriate indicator for identifying drought tolerance varieties (Fang and Xiong, 2015). In maize, the stay-green trait was considered to be closely related to yield (King and Purcell, 2001). Similar to leaf-rolling, the stay-green trait is traditionally evaluated using a manual visual score (Kholová and Vadez, 2013; Sukumaran et al., 2016).

The visual scores can only provide qualitative measurement of drought response. Most importantly, visual scores as evaluated by different people may be different, suggesting that the visual scores are subjective and error-prone. Continuous quantification of the drought response through measurement of leaf rolling and stay-green could, therefore, provide a useful means to monitor drought response. This would have particular advantages for large dispersed breeding trials.

Optical imaging and computer-assisted feature extraction have the advantages of being non-destructive, potentially high-throughput and objective. They have been widely applied in phenotyping plant growth and development (Spalding and Miller, 2013; Duan et al., 2015; Montagnoli et al., 2016; Rebolledo et al., 2016), seeds (Duan et al., 2011), root systems (Lobet et al., 2017), plant disease symptoms (Mutka and Bart, 2014), and plant abiotic stress (Altamimi et al., 2016; Fisher et al., 2016; Malinowska et al., 2017).

Honsdorf et al. (2014) calculated 8 imaging parameters, along with 3 harvest parameters and 4 indices, to evaluate the kinetics of growth under early drought stress and detect drought tolerance QTL in Barley. Petrozza et al. (2014) used non-invasive imaging to evaluate the plant water content, plant health state, state of the photosynthetic apparatus and digital biomass to characterize the response to drought stress in tomato. Born et al. (2014) adopted terahertz time-domain spectroscopy to measure the leaf water content for monitoring plant drought stress response. Stay-green or senescence was also calculated and used to evaluate drought responses (Petrozza et al., 2014; Malinowska et al., 2017). However, to the best of our knowledge, there is little research on quantification of leaf-rolling using these nondestructive approaches. Sirault et al. (2015) applied smoothing splines to skeletonized images of transverse wheat leaf sections to quantify the inter-genotypic variation for hydronastic leaf rolling in wheat. Neilson et al. (2015) estimated the degree of leaf rolling in sorghum by comparing projected plant area in the late afternoon (when the plants are least hydrated) with area early the following morning (when hydration would be expected to be maximal). They concluded that the decrease of projected leaf area was related to the degree of leaf-rolling over the diurnal cycle and the method seems effective over this time period. These and other related papers using pot experiments to study drought response and drought tolerance were listed in the Supplementary Presentation 1.

In this paper, we present a novel method to non-intrusively quantify drought response *in vivo* by analyzing simple color images of rice under both controlled and field conditions. Specifically, we

1. Provide an image analysis pipeline for analyzing images, extracting drought-related features that provide continuous and quantitative measurements related to the drought response,
2. Test the ability of the method to discriminate between drought resistant and drought sensitive accessions,
3. Test the ability of the method to dynamically quantify drought response under controlled and field conditions and finally
4. Show that the method can be easily adopted to quantify drought response and determine the initial date of leaf-rolling for *Miscanthus* and maize, indicating that the method is applicable to gramineous crops in general.

## Materials and methods

### Experimental design for dynamic quantification of rice drought response under controlled conditions

**Figure 1:**
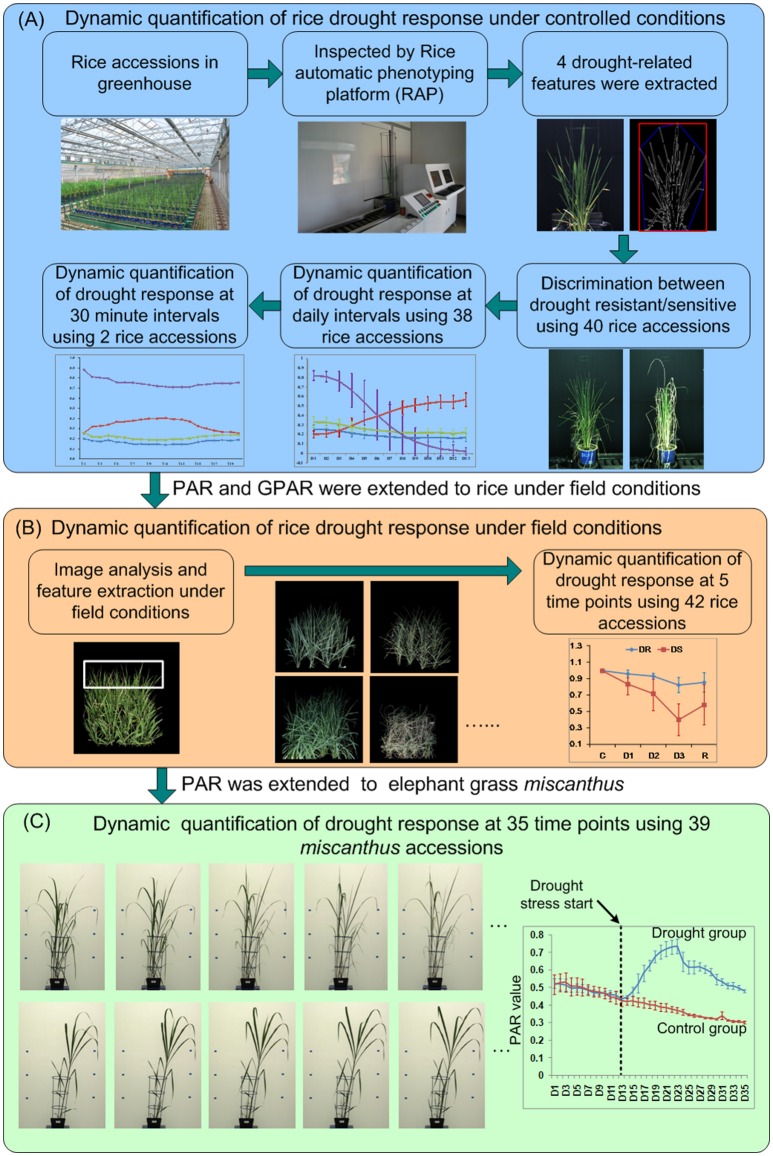
The experimental design for dynamic quantification of drought response for **(A)** rice under controlled conditions, **(B)** rice under field conditions, and **(C)** *Miscanthus* under controlled conditions.

The experimental design is shown in Figure 1A. To dynamically quantify rice drought response under controlled conditions, 40 accessions (20 drought resistant accessions and 20 drought sensitive accessions, Supplementary Presentation 2) with 4 replications of each genotype were grown in the greenhouse during summer, 2013. Each accession was deemed as drought resistant or drought sensitive by 3 experts evaluated manually based on its leaf-rolling and stay-green ability. The germination dates of the 40 accessions were staggered to ensure synchronized flowering. Seeds were sown in the field. Twenty-day-old seedlings were transplanted to pots containing 4.5 kg soil and transferred to a controlled environment. When an accession grew to the booting stage (panicle elongation), the plant was imaged by RAP (Rice Automatic Phenotyping, Yang et al., 2014) to collect image features before application of stress. Irrigation was then stopped to allow drought stress to occur. Soil water content was measured using TRIME-PICO32 (IMKO Micromodultechnik GmbH, Ettlingen, Germany) on the basis of time domain reflectometry (TDR). When the soil water content reduced to 15% (TDR value), the plants were watered to maintain the soil water content at 15% (TDR value) for 5 days. Then images of plants under stress conditions were collected using RAP. The time interval between the two imaging times was approximately 1 week, depending on the decline in soil water. The 4 image-derived features, whose behavior seemed related to drought response (1 feature related to stay-green and 3 shape descriptors, hereafter referred to as 4 drought-related features), were extracted and tested for their ability to discriminate known drought resistant accessions from drought sensitive accessions (see section Discrimination Between Drought Resistant and Drought Sensitive Accessions).

To further study the drought response from a dynamic standpoint, 38 accessions (Supplementary Presentation 2) that displayed pronounced leaf-rolling were planted at 18/August, 2015 as a single plants and subjected to drought stress from DAP 52 (days after planting) to DAP 70. All the plants were imaged daily by RAP from DAP 58 to DAP 70. The 4 drought-related features were extracted and analyzed to study the drought response at daily intervals. To study the detailed dynamics of the response to drought stress and rehydration, 2 accessions, namely *Maweinian* and *Zaoxian240* were monitored over the course of a day at approximately 30 min. intervals in summer, 2016. At the booting stage, irrigation was interrupted to allow drought stress to occur. While the soil remained visibly wet and no leaf-rolling occurred, images were taken to record the plants before stress. When the leaves of the plants started to roll, images were taken at approximately 30 min intervals to record the plants response under drought stress. After all the leaves of the plant rolled up, the plant was re-watered to collect images after rehydration at approximately 30 min intervals.

### Experimental design for dynamic quantification of rice drought response under field conditions

To test whether these drought-related features could be used to quantify drought response in the open field, 42 accessions with 2 replicates (Supplementary Presentation 2) were planted in an experimental paddy field (Figure 1B) in summer, 2016. Each accession was planted as a single 90 × 90 cm^2^ field-plot containing 20 plants (5 rows and 4 columns). A guard row was planted as a boundary between the field-plots to depress any edge effects. Images were taken with a consumer grade Nikon D40 camera. At the booting stage, irrigation was cut off to allow the drought stress to occur. Images were taken at five stages, which we classified as well watered (before stress, C), mild drought stress (D1), moderate drought stress (D2), severe drought stress (D3), and after rehydration (R). Specifically, when there was no water in the field but the soil kept wet (no visible leaf-rolling), images for plants before stress were collected. The time interval between different level of drought stress (D1 and D2, D2, and D3) was 5–6 days. Water was supplied again immediately after images under severe drought stress were taken. A week after rehydration, images for plants after rehydration were taken. Drought-related features were extracted from the images at all 5 stages to quantify the drought response.

### Experimental design for dynamic quantification of leaf-rolling in *Miscanthus*

To evaluate our method on other species grown on other phenotyping platforms, RGB side-view archival images from 39 accessions of the elephant grass *Miscanthus* were acquired from the large plant platform at National Plant Phenomics Centre (NPPC), IBERS, Aberystwyth University, UK (Malinowska et al., 2017). The 39 *Miscanthus* accessions (8 replicates for each accession) were planted at 07/ April, 2014, and moved to large plant platform at 12/ May, 2014. After transfer to NPPC, plants were grown for 2 weeks in well-watered conditions (90% relative soil water). Drought stress treatments were applied at roughly the time of emergence of the fifth leaf of the main stem. 4 replicates for each accession were treated for control (90% relative water capacity), and the other 4 replicates were treated for drought (taken to and held at 15–20% relative water capacity). Lateral side view RGB images (2 per day per plant) had been acquired daily from DAP 37 to DAP 71 and were re-used here to extract and calculate the drought related features (Figure 1C). Detailed experimental designs were listed in the Supplementary Presentation 3.

### Image capture and analysis under controlled conditions

Images of pot-grown rice plants were captured using the visible light imaging unit in the RAP (Yang et al., 2014) previously developed by our group. The RAP facility adopted an industrial conveyor to move the pot-grown rice plants to the imaging area. As the plants were rotated, side-view images from different angles were captured by a Charge Coupled Device (CCD) camera (Stingray F-504C, Applied Vision Technologies, Germany). Image acquisition was performed using NI-IMAQ Virtual Instruments (VI) Library for LabVIEW (National Instruments Corporation, USA). More details concerning the RAP system can be found in Yang et al. (2014).

**Figure 2:**
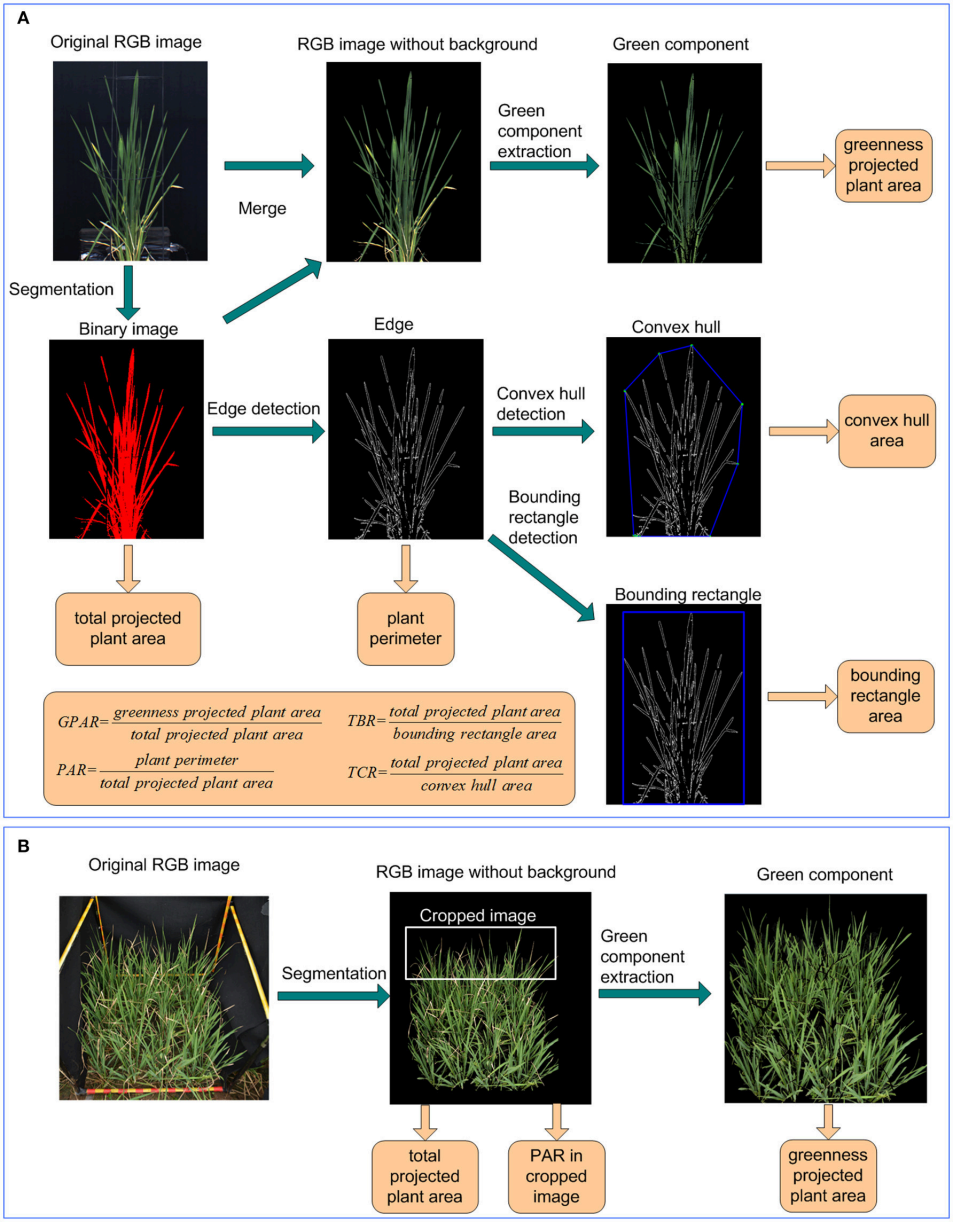
Image analysis and feature extraction pipeline under **(A)** controlled conditions and **(B)** field conditions.

The image analysis and feature extraction was performed in LabVIEW (National Instruments Corporation, USA). Image analysis consisted of 6 steps (Figure 2A):

1. Segmentation and total projected plant area calculation: the original RGB image was transformed to HSI color space. Background pixels and plant pixels were discriminated using fixed thresholds. A binary image of the plant was obtained by setting plant pixels as 1 and background pixels as 0. Regions with areas less than a predefined threshold were removed. The number of plant pixels was computed as total projected plant area.
2. Green component extraction and greenness projected plant area calculation: Using the binary image of the plant as a mask, a RGB image without background was generated from the original RGB image. The ExG and ExR planes of the plant's RGB image were extracted according to Equations (1, 2). The pixels were deemed as greenness pixels if their ExG value was greater than a predefined ExG threshold and their ExR value was less than a predefined ExR threshold. Greenness projected plant area was then calculated as the number of greenness pixels.
  $$\begin{array}{l} \text{ExG}=2\text{Ng}-\text{Nr}-\text{Nb} \end{array}$$
  $$\begin{array}{l} \text{ExR}=1.4\text{Nr}-\text{Nb} \end{array}$$
  where Nr, Ng, Nb was the normalized r, g, b plane, defined by Equations (3–5).
  $$\begin{array}{l} \text{Nr}=\text{R}/\left(\text{R}+\text{G}+\text{B}\right) \end{array}$$
  $$\begin{array}{l} \text{Ng}=\text{G}/\left(\text{R}+\text{G}+\text{B}\right) \end{array}$$
  $$\begin{array}{l} \text{Nb}=\text{B}/\left(\text{R}+\text{G}+\text{B}\right) \end{array}$$
  where R, G, B was the R, G, B plane of the RGB image, respectively.
3. Edge detection and plant perimeter calculation: The edge of the plant was extracted using IMAQ Edge Detection VI. The plant perimeter was calculated as the number of pixels in the plant edge.
4. Bounding rectangle detection and bounding rectangle area calculation: The bounding rectangle of the plant was the minimum rectangle that enclosed the plant. In this study, the bounding rectangle was detected using IMAQ Particle Analysis VI. The bounding rectangle area was calculated as the product of its width and height.
5. Convex hull detection and convex hull area calculation: The convex hull of the plant was the smallest convex set that contained the plant and detected using OpenCV. The convex hull area was computed as the pixel number of the convex hull.
6. Drought-related feature extraction: 4 image-derived features related to drought were calculated, including 1 stay-green related feature: greenness plant area ratio (GPAR), and 3 shape descriptors: total plant area/bounding rectangle area ratio (TBR), perimeter area ratio (PAR), and total plant area/convex hull area ratio (TCR). The definitions of the 4 drought-related features were provided in Equations (6–9).
  $$\begin{array}{l} GPAR=\frac{greenness\;projected\;plant\;area}{total\;projected\;plant\;area} \end{array}$$
  $$\begin{array}{l} TBR=\frac{total\;projected\;plant\;area}{bounding\;rectangle\;area} \end{array}$$
  $$\begin{array}{l} PAR=\frac{plant\;perimeter}{total\;projected\;plant\;area} \end{array}$$
  $$\begin{array}{l} TCR=\frac{total\;projected\;plant\;area}{convex\;hull\;area} \end{array}$$

These 4 drought-related features were extracted from each image. The drought-related features for a plant at a specific time point was then computed as the average feature value across all the side-view images from different angles at a given time point. The image analysis pipeline for *Miscanthus* was similar to the indoor rice, allowing for differences in number of side images.

### Image analysis for GPAR and PAR of rice under field conditions

Images of rice in field-plots were taken with a Nikon D40 camera containing a 23.7 × 15.6 mm CCD matrix of 6.24 million pixels, and equipped with a 35 mm focal length lens. As shown in Figure 2B, after original images were captured in field plot, the green component was extracted also using ExG plane. With the greenness projected plant area and total projected plant area in plot, the GPAR was calculated using the greenness projected plant area divided by total projected plant area. To avoid interference of overlapping plants and to better detect leaf-rolling, the top 1/3 of the original image was isolated from the whole image and used to calculate PAR. The detail of image analysis for GPAR and PAR under field conditions was shown in the Supplementary Presentation 4.

### Statistical analysis

Statistical analyses used SPSS (Statistical Product and Service Solutions, Version 19.0, SPSS Inc., USA). To test the effect of replications, genotypes and water treatment, the one-way analysis of variance (ANOVA) with subsequent *post-hoc* pairwise comparison using Tukey Honest Significant Difference (HSD) was applied at 95% confidence level. Pearson coefficients were calculated to analysis the relevance of the drought-related features under controlled conditions and field conditions.

## Results and discussion

### Discrimination between drought resistant and drought sensitive accessions

**Figure 3:**
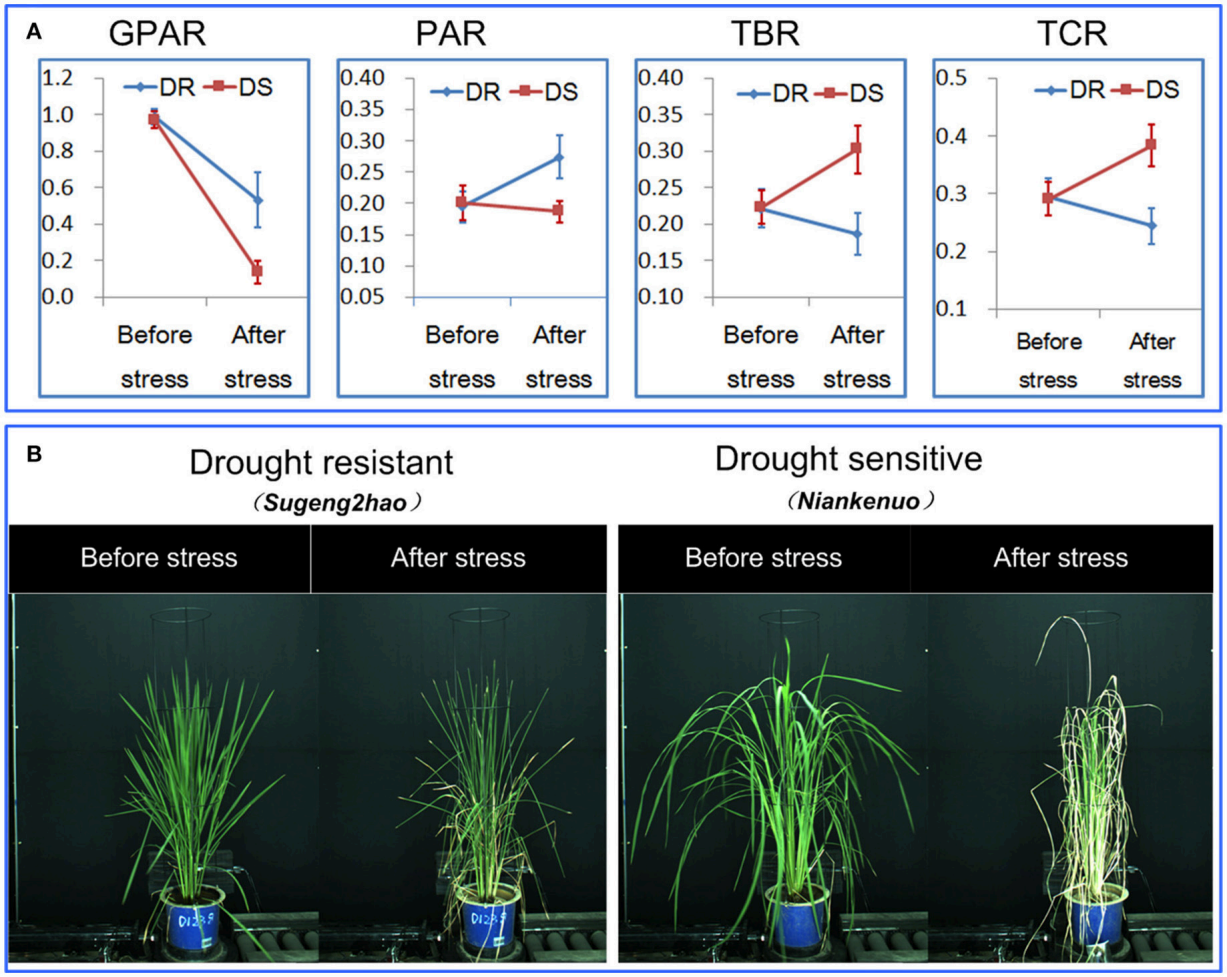
Discrimination between rice drought resistant and drought sensitive accessions.**(A)** General dynamics of 4 drought-related features before/after stress between drought resistant and drought sensitive accessions. The markers and the bars in each line represent the mean value and standard deviation across the accessions, respectively. **(B)** A drought resistant and a drought sensitive accession before and after stress.

To determine the ability of the 4 drought-related features in discriminating drought resistant accessions from drought sensitive accessions under controlled conditions, 40 accessions were subjected to drought stress. The digital features before and after drought stress were extracted. First, we tested whether there was a significant replication effect. There were no significant differences between the 4 replications (ANOVA, *p* > 0.05, Supplementary Table 1 in Supplementary Presentation 5). The mean of the 4 replications for each feature was then calculated and used for subsequent analysis. We analyzed the dynamics of the 4 drought-related features before/after stress between drought resistant and drought sensitive accessions (Figure 3). The markers and the bars in each line represent the mean value and standard deviation across the accessions, respectively. There were no significant differences between drought resistant and drought sensitive accessions for all 4 drought-related features before stress (ANOVA, *p* > 0.05, Supplementary Table 2 in Supplementary Presentation 5). In contrast, significant differences were observed between drought resistant and drought sensitive accessions for all 4 features after stress (ANOVA, *p*-value ranging from 2.261E-15 to 3.078E-12, Supplementary Table 2 in Supplementary Presentation 5). This indicated that the drought resistant and drought sensitive accessions responded to the drought treatment differently and that all the image derived features reported this difference faithfully.

More specifically, GPAR of both the drought resistant and drought sensitive accessions decreased after the drought stress. However, the decreasing amplitude of GPAR was much smaller for drought resistant accessions than drought sensitive accessions. PAR of the drought resistant accessions increased after drought stress while PAR of the drought sensitive accessions was relatively stable. In contrast to PAR, TBR and TCR decreased after drought stress in drought resistant accessions yet increased in drought sensitive accessions. These results show that the 4 drought-related features are capable of quantifying the differences in response to drought between drought resistant accessions and drought sensitive accessions.

**Figure 4:**
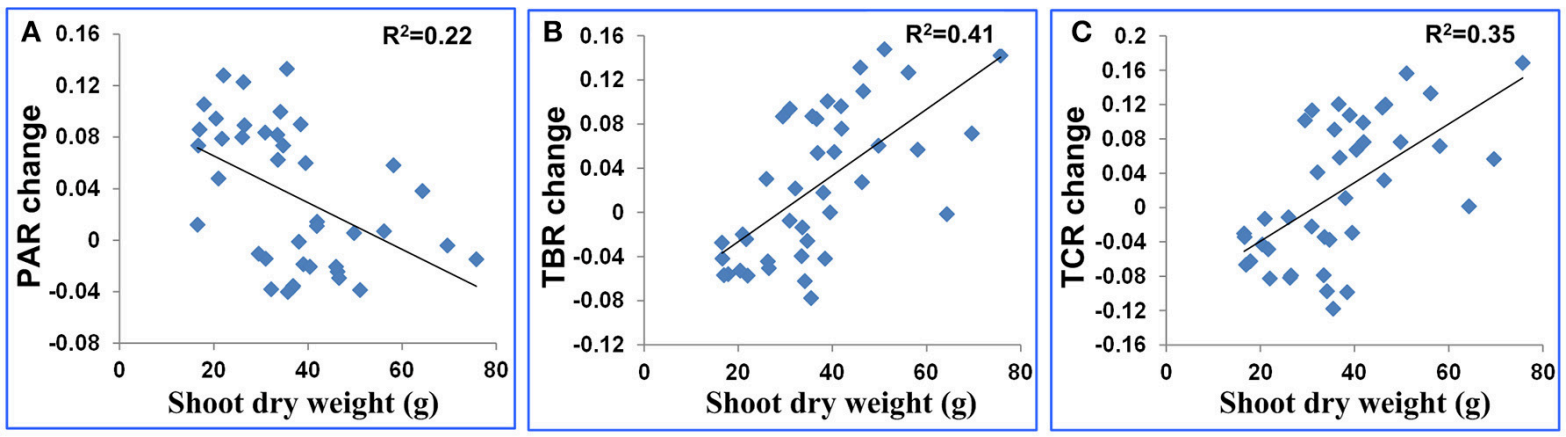
Scatter plot for plant dry weight after stress (g) and **(A)** the PAR change, **(B)** TBR change and **(C)** TCR change. The feature change was defined as feature value after stress minus feature value before stress.

**Figure 5:**
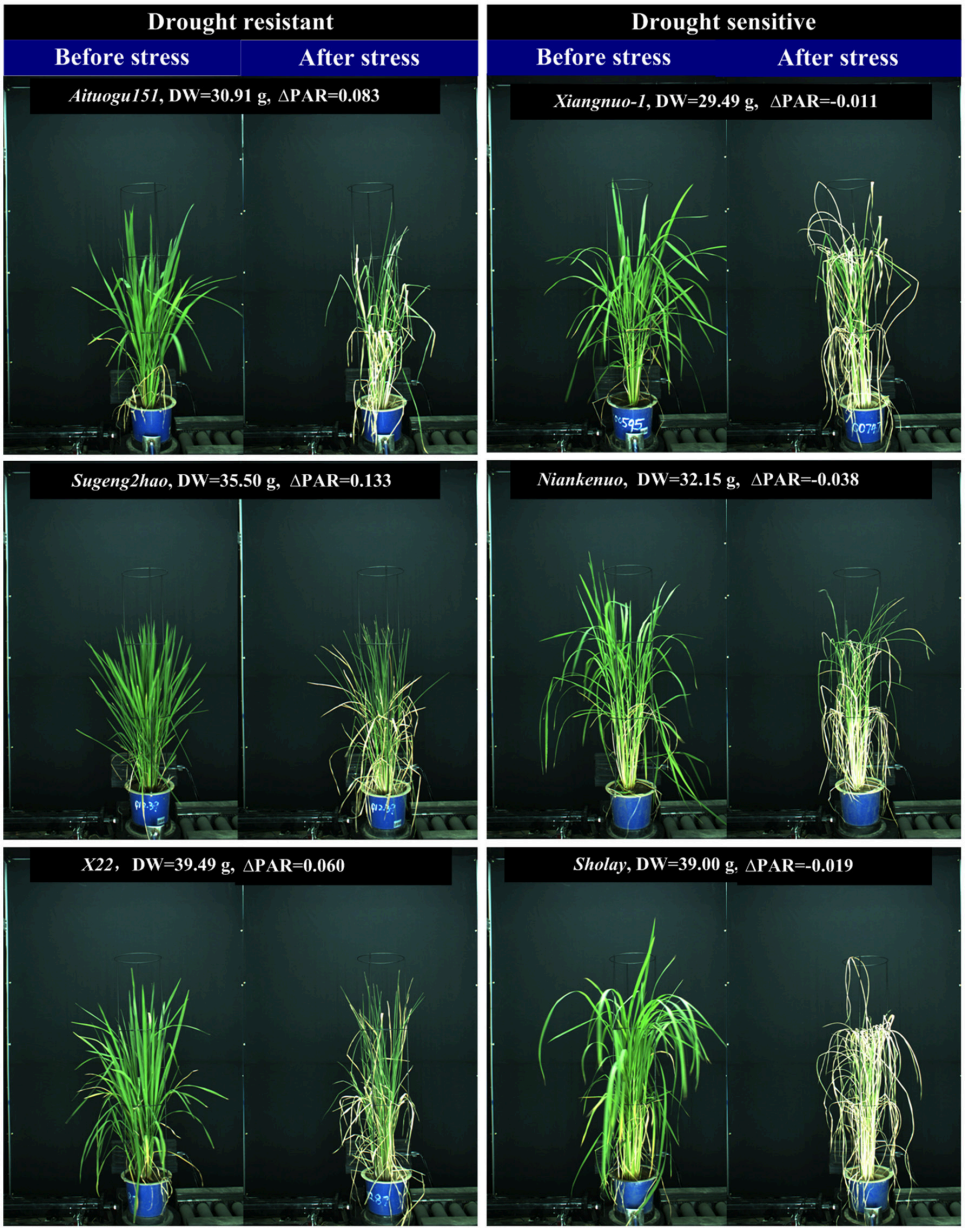
Accessions with similar plant dry weight may have different drought tolerance. ΔPAR was PAR change, and DW was plant dry weight after stress (g).

Plant size is a known variable that affects water use and potentially could be a confounding factor in the interpretation of these features. To estimate the extent to which plant size affected the features, we used TDR as a direct measure of soil water to impose water stress and then compared plants of comparable biomass. When we surveyed the entire population based on biomass (dry weight), there was only a weak relationship between shoot dry weight and PAR change (*R*^2^ = 0.22, Figure 4A). And there were moderate relationships between shoot dry weight and the other 2 shape descriptors (*R*^2^ were 0.41 and 0.35 for TBR change and TCR change, respectively, Figures 4B,C). The change of a drought-related feature was calculated as the feature value after stress minus the feature value before stress. Genotypes of comparable biomass could have either high scores or low scores (Figure 5). Therefore, we concluded that the features are unlikely to be directly related to plant size, and are more likely to be affected by actual water use and the ability of the leaves to conserve water.

### Dynamic quantification of drought response at daily and 30 minute intervals

The 38 accessions were monitored at daily intervals for 13 days to study the dynamic response to drought. Supplementary Figure 1A illustrates general dynamics of the 4 drought-related features for rice. Supplementary Figure 1B illustrates the dynamics of the 4 drought-related features for a randomly selected accession *PeiC12*2. In general, as the drought stress intensified, PAR increased while TBR, TCR and GPAR decreased, which was in agreement with theoretical predictions. Supplementary Figure 1C shows the dynamics of the first derivative of the 4 drought-related features for accession *PeiC122*. The first derivative of a specified feature was defined as X_i_- X_i−1_, where X_i_ was the feature value at the i-th day. From day 5(D5) to day 6(D6), the peak or valley of the first derivative of the 3 shape descriptors indicated visibly detectable leaf-rolling. As shown in Supplementary Figure 1C, visibly detectable leaf-rolling occurred at D6, which was consistent with Supplementary Figure 1D and Supplementary Video 1. Supplementary Figures 2 and 3 illustrate the detailed dynamics of the 4 drought-related features for accessions *Maweinian* and *Zaoxian240*, respectively. As expected, PAR increased as the drought stress intensified and then decreased after rehydration, while TBR, TCR and GPAR decreased as the drought stress intensified and increased after rehydration. An RGB image series for accession *Maweinian* at 30 min intervals is shown in Supplementary Figure 2B and Supplementary Video 2.

### Comparison of the 3 shape descriptors in quantification of leaf-rolling

**Figure 6:**
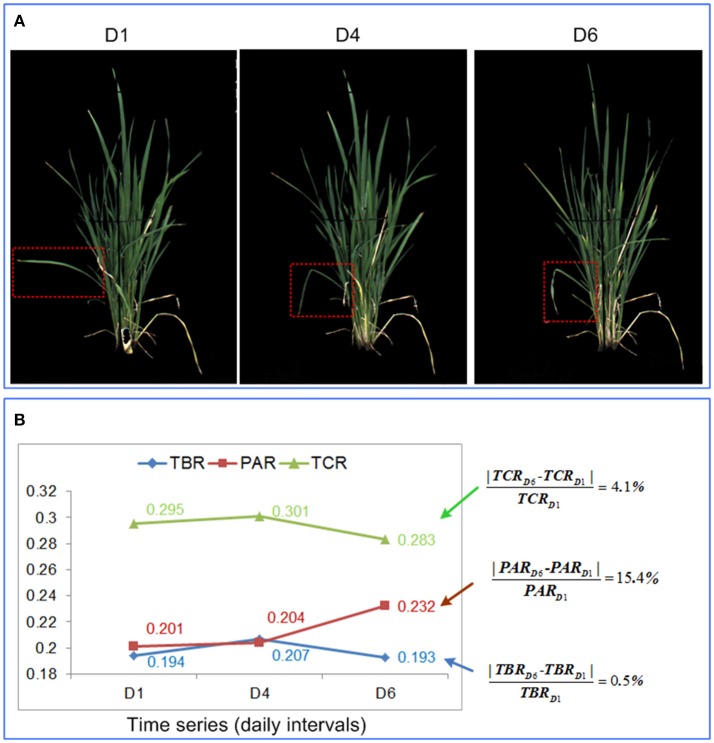
Comparison of the 3 shape descriptors in quantification of leaf-rolling. **(A)** RGB image at day 1, day 4, and day 6 for a rice plant. **(B)** Values of the 3 shape descriptors at day 1, day 4, and day 6, with the percentage change in each feature provided to the right.

As discussed in the previous sections, TBR, PAR, and TCR all provided appropriate and continuous measures of leaf-rolling when plants were subjected to drought. However, PAR tended to be more robust than TBR and TCR. As shown in Figure 6, theoretically, PAR should increase while TBR and TCR decrease as drought stress intensifies. However, TBR and TCR were more susceptible to perturbation by non-drought related factors, such as wind or cultivation induced damage. For example, in Figure 6A, a single leaf within the red dashed bounding rectangle became bent at day 4, probably due to mechanical damage. This reduced the size of the minimum bounding rectangle and the calculated convex hull. On the other hand, the reduction of the total projected plant area caused by leaf-rolling was relatively small. Therefore, instead of decreasing, TBR and TCR increased, in contrast to the aforementioned theoretical expectation. At day 6, the leaves started to roll, and TBR and TCR decreased as expected. However, the change rate of TBR and TCR were much smaller than PAR, indicating that PAR was more sensitive to leaf-rolling (Figure 6B). In addition, as shown in Figure 4, compared with TBR change and TCR change, PAR change had a weaker relationship with biomass, which indicated that PAR is less related to plant size. We conclude therefore that PAR is more robust and sensitive to leaf-rolling than either TBR and TCR. For these reasons, PAR was selected as the optimal digital proxy for leaf-rolling.

### Quantification of drought response under field conditions

**Figure 7:**
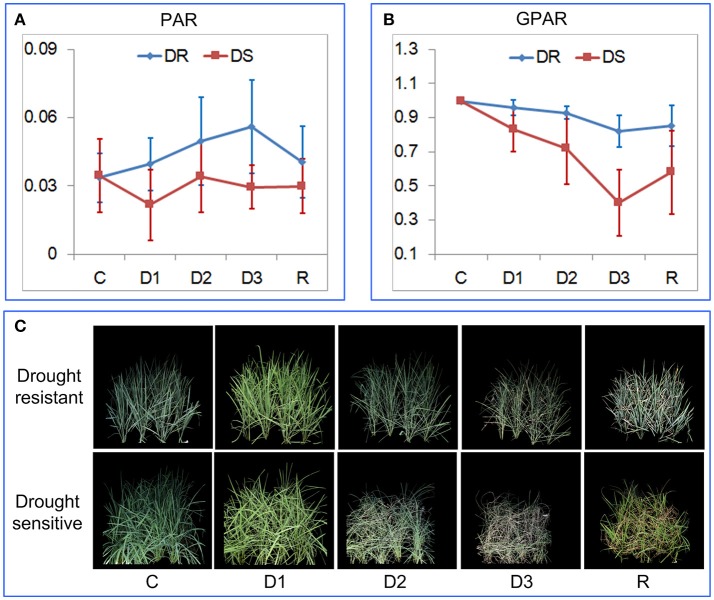
Quantification of rice drought response under field conditions. **(A)** Dynamics of PAR at five time points. **(B)** Dynamics of GPAR at five time points. The markers and the bars in each line represent the mean value and standard deviation across the accessions, respectively. **(C)** A drought resistant accession and a drought sensitive accession at five time points. C, before stress; D1, mild drought stress; D2, moderate drought stress; D3, severe drought stress, and R: after rehydration.

To verify the ability of PAR and GPAR in quantifying drought response under field conditions, 42 accessions were cultivated in field plots and subjected to drought stress. The time series of PAR and GPAR at five time points (C: before stress, D1: mild drought stress, D2: moderate drought stress, D3: severe drought stress, and R: after rehydration) were calculated and analyzed (Figure 7). First, we tested whether there was a significant replication effect. There were no significant differences between the replications (ANOVA, *p* > 0.05, Supplementary Table 3 in Supplementary Presentation 5). The mean of the replications for each feature was then calculated and used for subsequent analysis. There were no significant differences between drought resistant and drought sensitive accessions for both PAR and GPAR before stress (ANOVA, *p* > 0.05, Supplementary Table 4 in Supplementary Presentation 5). In contrast, significant differences were observed between drought resistant and drought sensitive accessions for PAR and GPAR at time points D1, D2, D3, R (ANOVA, p value ranging from 5.572E-12 to 0.029, Supplementary Table 4 in Supplementary Presentation 5). This suggested that drought resistant and drought sensitive accessions responded to the water restriction differently in a field environment. As shown in Figure 7A, PAR of the drought resistant accessions increased as the drought stress intensified and decreased after rehydration, while PAR of the drought sensitive accessions dropped after mild drought stress and was relatively stable as the drought stress intensified and after rehydration. As shown in Figure 7B, GPAR of both drought resistant and drought sensitive accessions decreased as the drought stress intensified and increased after rehydration. However, the amplitude of variation in GPAR was much smaller for drought resistant accessions than drought sensitive accessions.

### Correlation analysis of the drought-related features under controlled conditions and field conditions

We further examined whether the drought-related features derived under controlled conditions reflected, and were correlated with, those under field conditions. The same 42 rice accessions used in the field experiment (section: Quantification of Drought Response Under Field Conditions) with 4 replications were phenotyped under controlled conditions. The experimental design was the same as that described in section Experimental Design for Dynamic Quantification of Rice Drought Response Under Controlled Conditions. GPAR and PAR before and after stress were calculated and analyzed. Similarly, we first tested whether there was a significant replication effect. There were no significant differences between the 4 replicates (ANOVA, *p* > 0.05, Supplementary Table 5 in Supplementary Presentation 5). The mean of the 4 replicates for each feature was then calculated and used for subsequent analysis. There were no significant differences between drought resistant and drought sensitive accessions for GPAR and PAR before stress under controlled conditions (ANOVA, *p* > 0.05, Supplementary Table 6 in Supplementary Presentation 5). In contrast, significant differences were observed between drought resistant and drought sensitive accessions for all 4 features after stress (ANOVA, p value ranging from 4.862E-04 to 2.584E-03, Supplementary Table 6 in Supplementary Presentation 5).

**Figure 8:**
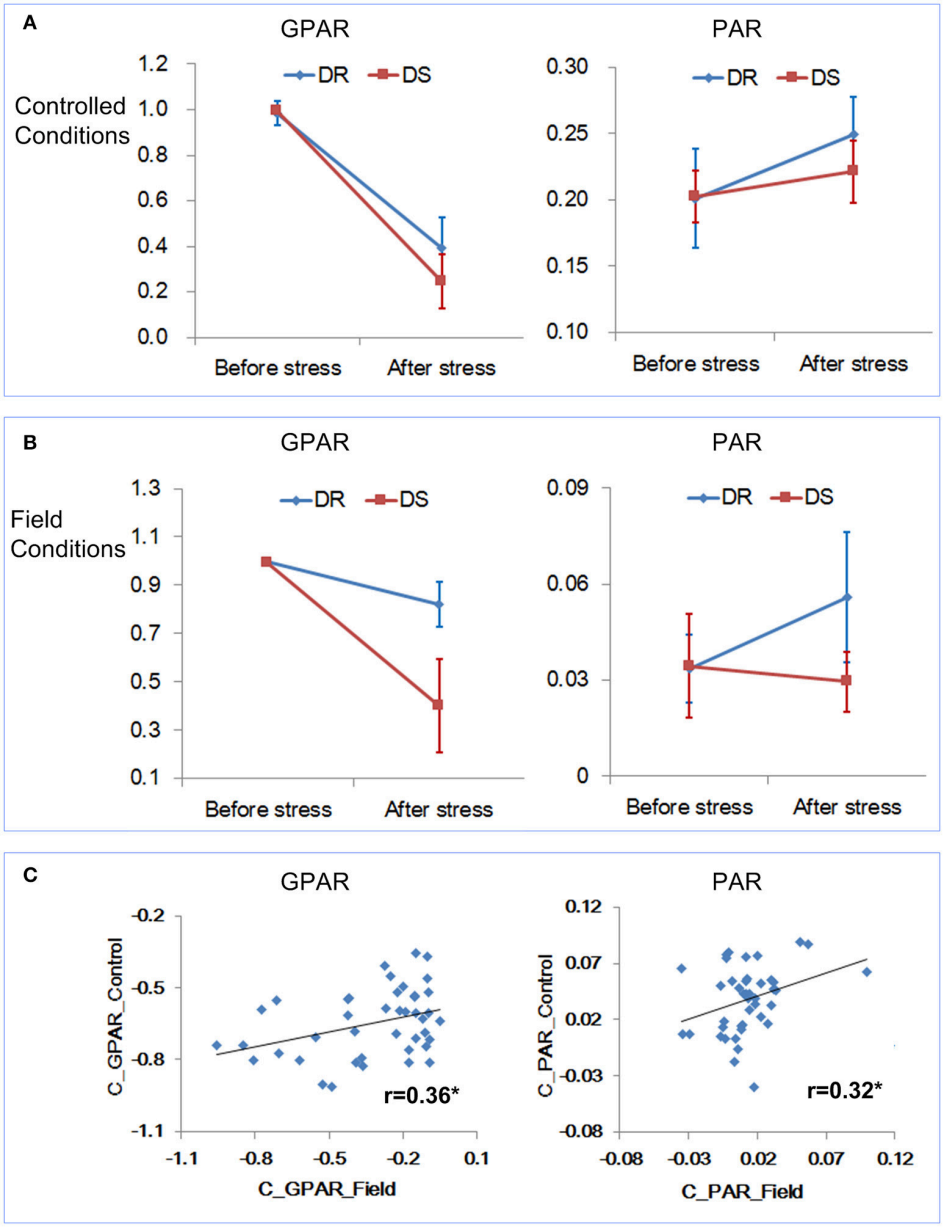
Relevance of the drought-related features under controlled conditions and field conditions. **(A)** Dynamics of GPAR and PAR under controlled conditions. **(B)** Dynamics of GPAR and PAR under field conditions. The markers and the bars in each line represent the mean value and standard deviation across the accessions, respectively. **(C)** Scatter plot of feature change under controlled conditions vs. feature change under field conditions.

First the change trends of GPAR and PAR both under controlled conditions and field conditions were compared. Because the rice accessions had gone through severe drought stress under controlled conditions, the time point D3 (severe drought stress) under field conditions was deemed as the corresponding time point of “after stress” under controlled conditions. As shown in Figures 8A,B, similar results were obtained under both controlled and field conditions. GPAR of both the drought resistant and drought sensitive accessions decreased after the drought stress. However, the decreasing amplitude of GPAR was much smaller for drought resistant accessions than drought sensitive accessions. PAR of the drought resistant accessions increased after drought stress while PAR of the drought sensitive accessions was relatively stable. Then we evaluated the correlation between feature changes under controlled conditions (represented by the suffix “_Control”) and feature changes under field conditions (represented by the suffix “_Field”). As is shown in Figure 8C and Supplementary Table 7 in Supplementary Presentation 5, feature change under field conditions exhibited a significant positive correlation with feature change under controlled conditions. In addition, to test the robustness of the correlation between controlled conditions and field conditions for GPAR change and PAR change, 40 rice accessions were randomly selected from the 42 rice accessions for the correlation analysis (10 repeated analysis). The results showed the range of r was 0.339–0.414 for GPAR change (Supplementary Presentation 6) and 0.315–0.371 for PAR change (Supplementary Presentation 7), respectively with all p values below 0.05. This indicated the robustness of the correlation of GPAR and PAR between controlled conditions and field conditions. We conclude, therefore, that GPAR and PAR can be extended to quantify drought response in field but the relatively low level of the correlation probably reflects root restriction in pots (Poorter et al., 2012).

### Quantification of drought response in other species using PAR

To test the generality of PAR as a means to quantify the leaf-rolling in other species grown on other phenotyping platforms, 39 *Miscanthus* accessions were imaged daily and analyzed. PAR values for the accessions grown under two water treatments (control and drought) were obtained over 35 time points. There were no significant differences between the 4 replicates (ANOVA, *p* > 0.05, Supplementary Table 8 in Supplementary Presentation 5). The mean of the 4 replicates for each feature was then calculated and used for subsequent analysis. There were no significant differences between the drought and control groups before time point D20 (ANOVA, *p* > 0.05, Supplementary Table 9 in Supplementary Presentation 5). Significant differences between drought and control groups was first observed at time point D21 (ANOVA, *p* < 0.05, Supplementary Table 9 in Supplementary Presentation 5) and highly significant differences appeared since time point D22 (ANOVA, *p*-value ranging from 3.066E-06 to 0.019, Supplementary Table 9 in Supplementary Presentation 5).

**Figure 9:**
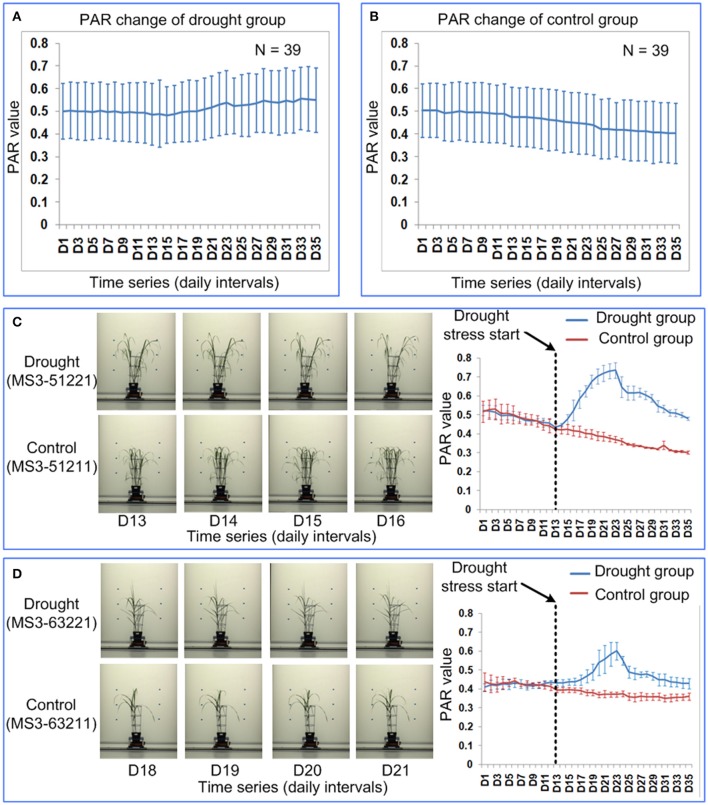
Quantification of leaf-rolling for 39 *Miscanthus* accessions at daily intervals. Dynamics of PAR at 35 time points for **(A)** drought group and **(B)** control group. The markers and the bars in each line represent the mean value and standard deviation across the accessions, respectively. **(C)** Leaf-rolling performance and PAR change for a *Miscanthus* accession under drought (MS3-51221) and control (MS3-51211). **(D)** Leaf-rolling performance and PAR change for a *Miscanthus* accession under drought (MS3-63221) and control (MS3-63211). **(C,D)** Illustrate the difference in leaf-rolling performance among two accessions and the difference in PAR change in drought/control group for each accession. The markers and the bars represent the mean value and standard deviation for the 4 replications, respectively.

In general, the PAR value of the control group decreases over the 35 time points (Figure 9B), because the *Miscanthus* plant grows rapidly and the growth rate of the plant perimeter is less than the growth rate of projected plant area. The PAR value of the drought group first decreased at a similar rate to the control and then increased, which indicated leaf-rolling (Figure 9A). The leaf-rolling performance for the drought group varied across the different *Miscanthus* accessions (Figures 9C,D). The leaf-rolling performance (such as initial date and variation rate of leaf rolling) may be related to the level of drought resistance. In addition, the ability of PAR to quantify leaf-rolling for maize plants was also demonstrated and a similar trend in PAR change can be obtained after drought treatment (Supplementary Presentation 8).

### Phenotyping drought response using non-destructive imaging

Non-destructive imaging was shown to be an effective method for measuring drought response in plant. Plant area, estimated from images, is considered as a proxy for biomass and plant growth, and has been adopted to evaluate and quantify the plant response to drought stress (Honsdorf et al., 2014; Petrozza et al., 2014; Fisher et al., 2016; Malinowska et al., 2017). However, as organ occlusion becomes more pronounced (for instance, for many cereals including rice and wheat at the reproductive stages), the accuracy of projected plant area for estimating the biomass and plant growth decreases (Munns et al., 2010).

Sirault et al. (2015) applied smoothing splines to skeletonized images of transverse wheat leaf sections to quantify inter-genotypic variation for hydronastic leaf rolling in wheat. Their method was destructive and involved sampling the flag leaves, excising a 30 mm segment that needs careful orientation prior to image capture under a dissecting microscope. Although effective, this method would be difficult to scale as each observation quantifies the leaf rolling of a single leaf at a single point in time. Neilson et al. (2015) quantified the effect of leaf rolling on the diurnal variation in leaf area by imaging plants in the late afternoon (when the plants are least hydrated) and early the following morning (when hydration would be expected to be maximal). They concluded that leaf rolling caused the decrease of leaf area and the degree of leaf-rolling could be estimated by the reduction of leaf area. Their method was efficient in quantification of leaf-rolling over the diurnal cycle. However, for long term quantification with intermittent imaging, this method may lose its efficacy because the plant area increases over time as the plant grows. We present a robust image feature PAR that is largely independent of imaging frequency, as well as being platform-agnostic. The method should therefore be applicable to quantifying responses to water restriction in a broad range of cereals and related grasses, under a wide range of environments.

## Conclusions

This paper demonstrates the use of high-throughput phenotyping and image analysis to dynamically quantify rice drought response *in vivo* in a non-intrusive manner. We propose a simple method for continuous measurement of drought response under controlled and field conditions. Three shape descriptors, namely, TBR, PAR, and TCR, were derived from the images and their behavior is related to changes in the leaf. We also present an image-derived feature, GPAR, for quantification of stay-green ability. Results showed that these 4 drought-related features were capable of discriminating drought resistant and drought sensitive accessions and dynamically quantifying the rice drought response under controlled conditions across time (at either daily or half hourly time intervals). We compared the 3 shape descriptors and concluded that PAR was more robust and sensitive to leaf-rolling than TBR and TCR. In addition, PAR and GPAR proved to be effective in quantification of drought response in the field. Notably, there was a small but significant correlation between drought responses of different genotypes in field and in pot, as measured by these features. This indicates that pot-based experiments can indeed provide an indication of drought responses of different genotypes, even though the physiology of drought is likely to be quite different under the 2 situations. Finally, we demonstrate the general applicability of the algorithms to other grass species, using our approach to test archival *Miscanthus* data that had previously been collected on an independent platform. In conclusion, this image-based technology provides a robust platform-independent tool for quantifying drought response and should be of general utility for breeding and functional genomics in future.

## Author contributions

LD and WY designed the research, performed the experiments, analyzed the data and wrote the manuscript. JH, FC, and KW performed the indoor grass experiments. JH and JD helped to write the manuscript. PY, DZ, and ZG performed the indoor rice experiments. HT performed the field-plot rice experiments. YF and MD performed the indoor maize experiments. GC provided the rice samples and also performed experiments. LX supervised the project and helped to design the research.

### Conflict of interest statement

The authors declare that the research was conducted in the absence of any commercial or financial relationships that could be construed as a potential conflict of interest.

## Supplementary material

Supplementary Video 1 RGB image series of accession *PeiC122* at daily intervals.

Supplementary Video 2 RGB image series of accession *Maweinian* at 30 minute intervals.

Supplementary Figure 1 Quantification of rice drought response at daily intervals. **(A)** General dynamics of 4 drought-related features for rice. The markers and the bars in each line represent the mean value and standard deviation across the accessions, respectively. **(B)** Time series of 4 drought-related features for a randomly selected accession *PeiC122*. **(C)** Time series of the first derivative of 4 drought-related features for accession *PeiC122*. **(D)** RGB image of accession *PeiC122* at day 5, 6, and 7.

Supplementary Figure 2 Quantification of response to drought and rehydration at 30 min intervals using rice accession *Maweinian*. **(A)** Time series of drought-related features. **(B)** RGB image at time points T1, T4, T11, and T20.

Supplementary Figure 3 Quantification of plant's response to drought and rehydration at 30 min intervals using rice accession *Zaoxian240*.

Supplementary Presentation 1 The related papers using pot experiments to study drought response and drought tolerance.

Supplementary Presentation 2 The detail of accessions used in this paper.

Supplementary Presentation 3 Experimental designs in this study.

Supplementary Presentation 4 The detail of image analysis for rice under field conditions.

Supplementary Presentation 5 Results of statistical analysis in this study.

Supplementary Presentation 6 The correlation analysis between controlled conditions and field conditions for GPAR change (10 repeated analysis).

Supplementary Presentation 7 The correlation analysis between controlled conditions and field conditions for PAR change (10 repeated analysis).

Supplementary Presentation 8 The details about the maize experiment and results.
