# Supplementary figures and images for: Novel Digital Features Discriminate Between Drought Resistant and Drought Sensitive Rice Under Controlled and Field Conditions
Source: Front Plant Sci. 2018 Apr 17;9:492. doi: 10.3389/fpls.2018.00492 (PMC5913589; doi:10.3389/fpls.2018.00492)

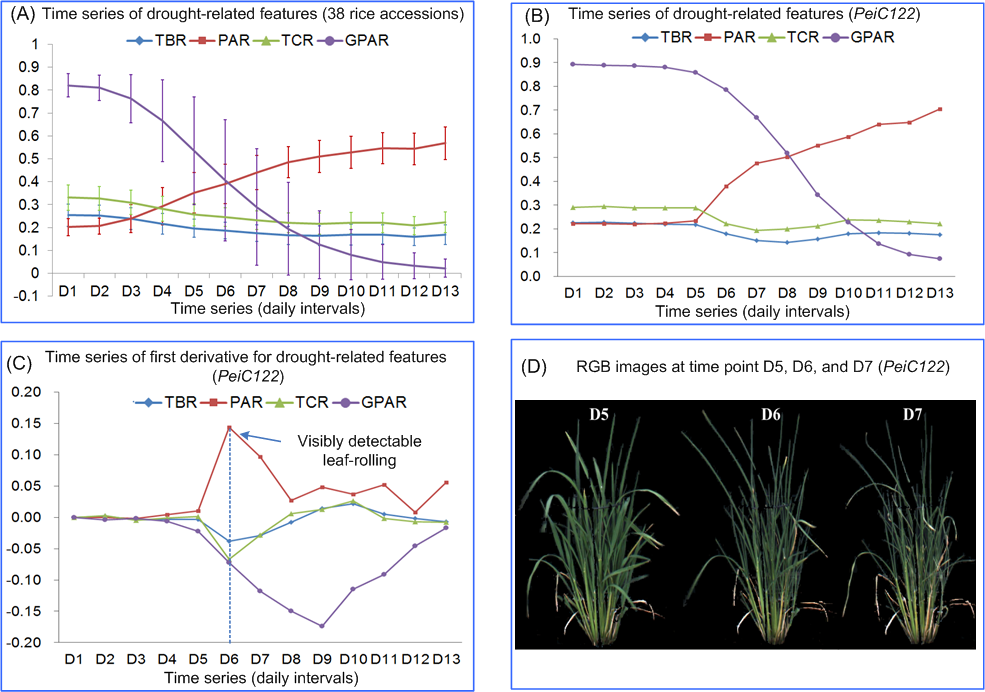

Supplement: Supplementary Figure 1 — Quantification of rice drought response at daily intervals. (A) General dynamics of 4 drought-related features for rice. The markers and the bars in each line represent the mean value and standard deviation across the accessions, respectively. (B) Time series of 4 drought-related features for a randomly selected accession PeiC122. (C) Time series of the first derivative of 4 drought-related features for accession PeiC122. (D) RGB image of accession PeiC122 at day 5, 6, and 7. [file Image1.TIF]

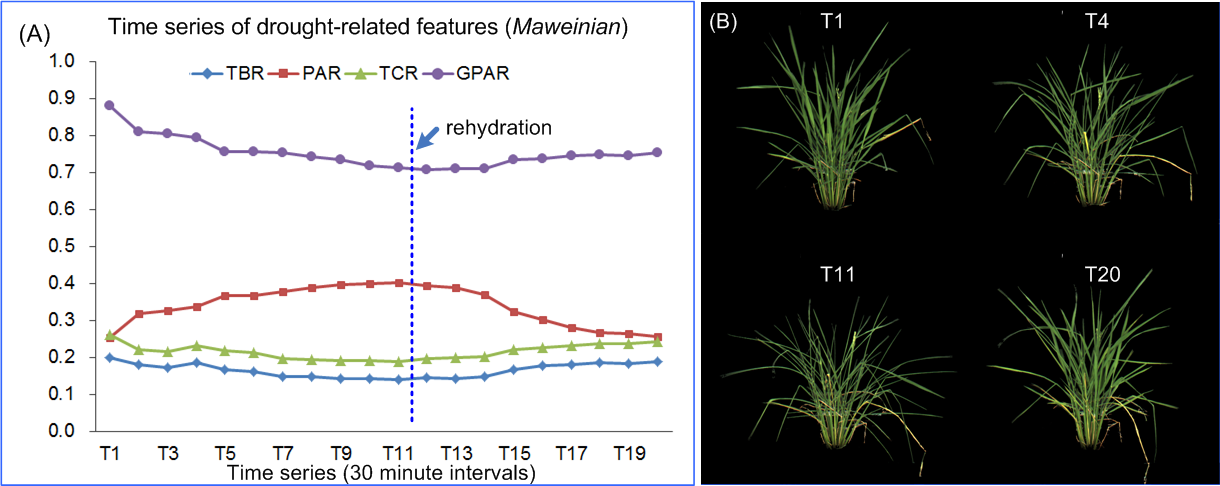

Supplement: Supplementary Figure 2 — Quantification of response to drought and rehydration at 30 min intervals using rice accession Maweinian. (A) Time series of drought-related features. (B) RGB image at time points T1, T4, T11, and T20. [file Image2.TIF]

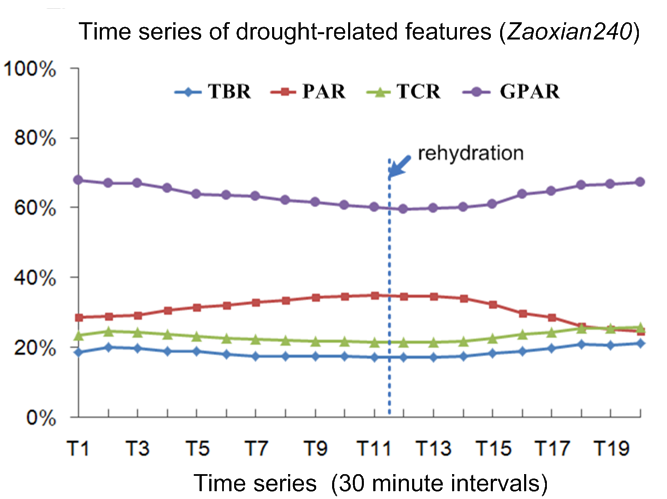

Supplement: Supplementary Figure 3 — Quantification of plant's response to drought and rehydration at 30 min intervals using rice accession Zaoxian240. [file Image3.TIF]
