# Supplementary material for: Novel Digital Features Discriminate Between Drought Resistant and Drought Sensitive Rice Under Controlled and Field Conditions
Source: Front Plant Sci. 2018 Apr 17;9:492. doi: 10.3389/fpls.2018.00492 (PMC5913589; doi:10.3389/fpls.2018.00492)
Supplement: Supplementary Presentation 1 — The related papers using pot experiments to study drought response and drought tolerance. [file Presentation1.PDF]

Supplementary Table 1 The related papers using pot experiments to study drought response and drought tolerance

Species	Number of genotypes	Pot size	Timing of drought stress	Parameters measured	Application	Reference
Rice	2	20cm diameter × 25cm height	2-3 weeks after transplanting, mid-tillering, panicle initiation and first flowering	leaf rolling score, transpiration rates, soil water status, leaf area index, early leaf senescence, dates of flowering, grain yield, yield components	Study the physiological and morphological responses to transient drought	Wopereis et al., 1996
Rice	5	20 L	vegetative stage reproductive stage	relative leaf water content, panicle lengths, days to flowering, days to maturity, yield at 14% moisture content, filled grain ratio percentage, grain yield	Evaluate the responses to water deficit	Sikuku et al., 2012
Rice	4	7.5Kg soil	vegetative stage reproductive stage	normalized transpiration rate, fraction of transpirable soil water	Compare the response of normalized transpiration rate (NTR) of the rice cultivars to soil water deficit	Heinemann et al., 2011
Rice	12	15cm diameter × 30cm height	booting stage	relative water content, proline content in the leaf blade, photosynthetic pigments, chlorophyll florescence, net-photosynthetic rate, transpiration rate, stomatal conductance in flag leaf and panicle traits	Water-deficit tolerant classification in mutant lines of indica rice	Chaum et al., 2012
Rice	2	-	seedling stage	leaf phenotype, survival rate, water loss	Identify a drought-hypersensitive mutant of a putative MAPK kinase gene	Jing et al., 2010
Barley		2.5 kg quartz sand	tillering stage	chlorophyll content, shoot biomass, root biomass, protein concentration	Study the influence of 2-aminoethanol on growth and yield of barley under different water supply	Mascher et al., 2005
Barley	37	25cm diameter × 25cm height	Vegetative stage reproductive stage	shoot dry weight, root dry weight ,plant height, number of fertile tiller, and relative water content root length, root volume	Access the response of barley to drought stress.	Barati et al., 2015
Sorghum bicolor L. Moench	2	15cm diameter × 20cm height	eight-leaf developmental stage	leaf area, height, shoot dry weight, leaf dry weight, sheath dry weight, leaf mass per unit area, dry matter content, relative growth rate, moisture content, water use efficiency, plant architecture, leaf rolling and senescence	Examine the dynamic phenotypic responses to water deficiency over time	Neilson et al., 2015
Sunflower	4	10cm diameter × 35cm height	5 days after the initiation of the eighth leaf	leaf area, soil water depletion rates	Evaluate drought tolerance of sunflower	Pereyra-Irujo et al., 2007
Cowpea	14	-	seedling stage	maintenance of stem greenness, unifoliate senescence, wilting, trifoliate abscission, and nodal anthocyanin accumulation	Investigate genotypic and phenotypic responses to seedling stage drought and identify drought-related genetic elements	Muchero et al., 2008

Cotton	3	3.6 L, 13 L	seven to eight leaf stage	biomass, transpirational water loss, relative water content, electrolyte leakage, lipid peroxidation, proline content	Test the function and potential use of *atlos5* gene in improving drought tolerance of cotton	Yue et al., 2012
Cotton	2	55cm diameter × 25cm height	five to six leaf stage	morphological traits (biomass, root and lateral root length, leaf number),physiological and biochemical characters	Test the function and potential utility of *tamnsod* for improving the drought tolerance of cotton	Zhang et al., 2014
Soybean	3	1.0 Kg<br>3.0 kg	V3 stage<br>V_4 stage	biomass, total transpiration, transpiration efficiency, relative growth rate, number of leaves, leaf area, height, and length of the internode	Phenotype soybean plants transformed with rd29a:Atdreb1a for drought tolerance	Rolla et al., 2014
Blackcurrant	2	19cm diameter × 14.9cm height	beginning of flowering	plant water relations, root growth, root activity , root length and diameter, leaf area and plant biomass	Study the drought stress tolerance of blackcurrant and ability to recover drought stress	Čereković et al., 2013
Miscanthus	47	22.5cm diameter × 17.8cm height	emergence of the fifth leaf of the main stem	biomass, stem number ,maximum tiller height, water use efficiency	Phenomics analysis of drought responses in miscanthus	Malinowska et al., 2017
European beech	11	17 cm diameter and 2 L volume	the first stage of shoot growth	water relations, abscisic acid, proline and sucrose	Identify drought-sensitive beech ecotypes	Peuke et al., 2002
Brachypodium distachyon	138<br>49	small pots (7.5cm diameter × 9cm height)<br>square pots (9 cm×9 cm×7.5 cm)	28 days after sowing<br>33 days after sowing	phenomic parameters linked to growth and color changes	Study natural variation in drought responses of brachypodium distachyon by combining dynamic phenotyping with metabolite analysis	Fisher et al., 2016
Barley	48	2.5 L	vegetative stage	8 imaging parameters, 3 harvest parameters and 4 Indices	Detect drought tolerance QTL using non-destructive high-throughput imaging to measure drought response in barley	Honsdorf et al., 2014
Barley	2	5cm diameter × 5cm height	5 leaf stage	water loss rate, survival rate	Elucidate the specific gene networks and pathways that contribute to drought tolerance, and identify new candidate genes	Liang et al., 2017
Wheat	150	2.5 L	3 weeks after sowing to flowering	soil water content, leaf water potential, plant weight, leaf area, biomass, growth rate, leaf expansion rate, water use efficiency	Identify the genetic control of growth and transpiration underlying yield response to water-deficit stress	Parent et al., 2015
Barley	49	1.5L	ten days post-anthesis until maturity	days to heading, plant height, yield parameters, plant biomass	Identify useful phenotypic variation for terminal drought tolerance by evaluating the drought response	Honsdorf et al., 2017

Tomato	-	16 cm diameter × 20 cm height	after the third or fourth leaf, non-cotyledon, had fully formed and continued until the end of the experiment	digital biomass, high water content index, stress index	Evaluate the impact of Megafol® in attenuating the negative physiological responses of drought	Petrozza et al., 2014
Silver fir	-	-	Seedling	fresh weight, dry weight, relative water content, and transmission	Monitoring plant drought stress response using terahertz time-domain spectroscopy	Born et al., 2014

References

1. Barati, M., Majidi, M. M., Mirlohi, A., Pirnajmodini, F., & Sharifmoghaddam, N. (2015). Response of cultivated and wild barley germplasm to drought stress at different developmental stages. Crop Science, 55(6), 2668-2681.
2. Born, N., Koch, M. , Liepelt, S., Beyer, S., Schwerdtfeger, M., Ziegenhagen, B., & Koch, M. (2014). Monitoring plant drought stress response using terahertz time-domain spectroscopy. Plant Physiology, 164(4), 1571-1577.
3. Čereković, M. Pagter, Kristensen, H. L., Pedersen, H. L., R. Brennan, & K.K. Petersen. (2013). Effects of drought stress during flowering of two pot-grown blackcurrant (ribes nigrum, l.) cultivars. Scientia Horticulturae, 162(3), 365-373.
4. Yooyongwech, S., & Supaibulwatana, K. (2012). Water-deficit tolerant classification in mutant lines of indica rice. Scientia Agricola, 69(2), 135-141.
5. Fisher, L.H., Han, J., Corke, F.M., Akinyemi, A., Didion, T., Nielsen, K.K., Doonan, J.H., Mur, L.A., and Bosch, M. (2016). Linking Dynamic Phenotyping with Metabolite Analysis to Study Natural Variation in Drought Responses of Brachypodium distachyon. Front Plant Sci 7, 1751.
6. Heinemann, A. B., Stone, L. F., & Fageria, N. K. (2011). Transpiration rate response to water deficit during vegetative and reproductive phases of upland rice cultivars resposta da taxa de transpiração ao déficit hídrico nas fases vegetativa e reprodutiva de cultivares de arroz de terras altas. Scientia Agricola, 68(1), 24-30.
7. Honsdorf, N., March, T. J., Berger, B., Tester, M., & Pillen, K. (2014). High-throughput phenotyping to detect drought tolerance QTL in wild barley introgression lines. Plos One, 9(5), e97047.
8. Honsdorf, N., March, T. J., & Pillen, K. (2017). Qtl controlling grain filling under terminal drought stress in a set of wild barley introgression lines. Plos One, 12(10), e0185983.
9. Jing, N., Li, X. H., Hicks, L. M., & Xiong, L. Z. (2010). A raf-like mapkkk gene dsm1 mediates drought resistance through reactive oxygen species scavenging in rice. Plant Physiology, 152(2), 876.
10. Liang, J., Chen, X., Deng, G., Pan, Z., Zhang, H., & Li, Q., et al. (2017). Dehydration induced transcriptomic responses in two tibetan hulless barley (hordeum vulgare var. nudum) accessions distinguished by drought tolerance. Bmc Genomics, 18(1), 775.
11. Malinowska, M., Donnison, I. S., & Robson, P. R. H. (2017). Phenomics analysis of drought responses in miscanthus collected from different geographical locations. Global Change Biology Bioenergy, 9(1),78-91.
12. Mascher, R., Nagy, E., Lippmann, B., Hörnlein, S., Fischer, S., & Scheiding, W., et al. (2005). Improvement of tolerance to paraquat and drought in barley (hordeum vulgare, l.) by exogenous 2-aminoethanol: effects on superoxide dismutase activity and chloroplast ultrastructure. Plant Science, 168(3), 691-698.
13. Muchero, W., Ehlers, J. D., & Roberts, P. A. (2008). Seedling stage drought-induced phenotypes and drought-responsive genes in diverse cowpea genotypes. Crop Science, 48(2), 541-552.

14. Neilson, E. H., Edwards, A. M., Blomstedt, C. K., Berger, B., Møller, B. L., & Gleadow, R. M. (2015). Utilization of a high-throughput shoot imaging system to examine the dynamic phenotypic responses of a c4 cereal crop plant to nitrogen and water deficiency over time. Journal of Experimental Botany, 66(7), 1817.
15. Parent, B., Shahinnia, F., Maphosa, L., Berger, B., Rabie, H., & Chalmers, K., et al. (2015). Combining field performance with controlled environment plant imaging to identify the genetic control of growth and transpiration underlying yield response to water-deficit stress in wheat. Journal of Experimental Botany, 66(18), 5481-5492.
16. Pereyra-Irujo, G. A., Velázquez, L., Granier, C., & Aguirrezábal, L. A. N. (2007). A method for drought tolerance screening in sunflower. Plant Breeding, 126(4), 445–448.
17. Petrozza, A., Santaniello, A., Summerer, S., Tommaso, G. D., Tommaso, D. D., & Paparelli, E., et al. (2014). Physiological responses to Megafol ®treatments in tomato plants under drought stress: a phenomic and molecular approach. Scientia Horticulturae, 174, 185-192.
18. Peuke, A. D., Schraml, C., Hartung, W., & Rennenberg, H. (2002). Identification of drought-sensitive beech ecotypes by physiological parameters. New Phytologist, 154(2), 373-387.
19. Rolla, A. A. D. P., Fuganti-Pagliarini, R., Engels, C., Rio, A. D., Marin, S. R. R., & Oliveira, M. C. N. D., et al. (2014). Phenotyping soybean plants transformed with rd29a:atdreb1a for drought tolerance in the greenhouse and field. Transgenic Research, 23(1), 75-87.
20. Sikuku P.A., Onyango J.C. & Netondo G.W.(2012). Yield Components and Gas Exchange Responses of Nerica Rice Varieties (Oryza Sativa L.) to Vegetative and Reproductive Stage Water Deficit. Global Journal of Science Frontier Research Agriculture & Biology,12(3), 50-62.
21. Wopereis, M. C. S., Kropff, M. J., Maligaya, A. R., & Tuong, T. P. (1996). Drought-stress responses of two lowland rice cultivars to soil water status. Field Crops Research, 46(1-3), 21-39.
22. Yue, Y., Zhang, M., Zhang, J., Tian, X., Duan, L., & Li, Z. (2012). Overexpression of the atlos5 gene increased abscisic acid level and drought tolerance in transgenic cotton. Journal of Experimental Botany, 63(10), 3741.
23. Zhang, D. Y., Yang, H. L., Li, X. S., Li, H. Y., & Wang, Y. C. (2014). Overexpression of tamarix albiflonum tamnsod increases drought tolerance in transgenic cotton. Molecular Breeding, 34(1), 1-11.
