# Supplementary material for: Novel Digital Features Discriminate Between Drought Resistant and Drought Sensitive Rice Under Controlled and Field Conditions
Source: Front Plant Sci. 2018 Apr 17;9:492. doi: 10.3389/fpls.2018.00492 (PMC5913589; doi:10.3389/fpls.2018.00492)
Supplement: Supplementary Presentation 2 — The detail of accessions used in this paper. [file Presentation2.PDF]

1. The names of indoor-growth 40 rice accessions used in discriminating drought resistant accessions and drought sensitive accessions. (Genotype data of rice is available in the website: http://ricevarmap.ncpgr.cn/snp_id/)

20 drought sensitive accessions*: Ak Tokhum, Lengshuigu, Spin Mere, UZ ROS 7-13, Thang 10 , Pathma wee, Minghui63, Niankenuo, Chun 118-33, GPNO 1106, Jhona349, Sholay, PHUDUGEY, Karayal, Xiangnuo-1, Karnal Local, Pengshantieganzhan, Mengguandamagu, Zegu, Leihuozhan*

20 drought resistant accessions*: X22, Sugeng2hao, La110, PeiC122, Zhongchao123, EMBRAPA 1200, CDR22, Gayabyeo, IR58025B, Aituogu151, Huajingxian74, MOROBEREKAN, TEQING, NEDA, Yuexiangzhan, Gang46B, Nipponbare, TGMS29, zhenshan97B-1, IR 2061-214-2-3*

2. The names of indoor-growth 38 rice accessions used in quantification of rice drought response at daily intervals.

Yuyannuo-1, Yuyannuo-2, Yunjiang35, PSBRC82, NSICRC122, X22, Niankenuo, Tsao wan ching, THAVALU, Aihechi, PeiC122, Tamanishiki, C71, IR 2071-625-1-252, Zhongchao123, EMBRAPA 1200, CDR22, Gayabyeo, CYPRESS, Aituogu151, Huajingxian74, Aichi Asahi, MOROBEREKAN, TEQING, IRAT109, NEDA, Yuexiangzhan, Wudadaozhong, Mayang Khang, Fengaizhan, CHOROFA, Nipponbare, BRRI DHAN 28, Basmati370, Zhenshan97B, IR 2061-214-2-3, NAN-29-2, AKITAKOMACHI.

3. The names of 42 rice accessions used in quantifying the drought response under field conditions

27 drought resistant accessions: *PSBRC82, X22, ASD16, PSB RC 66, Gu154, C71, Bg94-1, Xiushui115, Yuanjing7, Lemont, CM1, HAIPONG, WC 3532, Huajingxian74, Ai-Zi-DAO, Q5, AGNO (PSBRC28), OM 2517, Shan-Huang Zhan-2-1 , IRAT109, DNJ 179, Liaogeng287, M3122, TKM9, Gang46B, Huangkezaonian, Fengaizhan, Basmati370.*

15 drought sensitive accessions: *E B Gopher, Zaoshunonghu6, Ittikulama, Pengshantieganzhan, Guichao2hao, Mamagu-1, XiangaiB, Xibaizhan, Zinuo, A 152, 4595, AMANE, Zaoshuxianghei, Bombilla, Eiko.*
