# Supplementary material for: Novel Digital Features Discriminate Between Drought Resistant and Drought Sensitive Rice Under Controlled and Field Conditions
Source: Front Plant Sci. 2018 Apr 17;9:492. doi: 10.3389/fpls.2018.00492 (PMC5913589; doi:10.3389/fpls.2018.00492)
Supplement: Supplementary Presentation 3 — Experimental designs in this study. [file Presentation3.PDF]

Supplementary Table 1 Experimental designs in this study.

Purpose	Species	Indoor/outdoor phenotyping	Samples	Drought treatment	Duration of inspection	Inspection interval
To discriminate DR and DS accessions using 4 drought-related features	Rice	Indoor (RAP*)	40 accessions with 4 replications (20 drought resistant accessions and 20 drought sensitive accessions)	Irrigation was stopped to allow drought stress to occur at the booting stage (panicle elongation). When the soil water content reduced to 15% (TDR value), the plants were watered to maintain soil water content at 15% (TDR value) for five days.	Before stress<br>After stress	Approximately 1 week, depending on the declining rate of soil water
To dynamically quantify rice drought response under controlled conditions using 4 drought-related features	Rice	Indoor (RAP*)	38 accessions under drought treatment	Planted at 18/August, 2015 and no irrigation from DAP 52 to DAP 70	DAP 58 to DAP 70 (13 days)	Daily intervals
	Rice	Indoor (RAP*)	2 accessions under drought treatment	At the booting stage, water was cut off to allow the drought stress to occur. After all the leaves of the plant rolled up, the plant was re-watered.	1 day	Approximately 30 minutes
To extend the drought-related features to field	Rice	Field plot	42 accessions with 2 replications. Each accession was planted in a 90×90 cm^2	At the booting stage, water was cut off to allow the drought stress to occur. When there was no	Before stress (C),<br>mild drought stress (D1),	5-7 days

			field-plot containing 20 plants (5 rows and 4 columns).	water in the field but the soil kept wet (no visible leaf-rolling), images for plants before stress were collected. The time interval between different level of drought stress (D1 and D2, D2 and D3) was 5-6 days. Water was supplied again immediately after images under severe drought stress were taken. A week after rehydration, images for plants after rehydration were taken.	moderate drought stress (D2),<br>severe drought stress (D3),<br>and after rehydration (Re)	
To extend the drought-related features could be used to quantify drought response of other species grown on other phenotyping platforms	*Miscanthus*	Indoor (NPPC*)	39 accessions with 8 replications (4 replications with drought condition and 4 replications with control condition)	After transfer to NPPC, plants were grown for 2 weeks in well-watered conditions (90% relative soil water). Drought stress treatments were applied at roughly the time of emergence of the fifth leaf of the main stem. 4 replications were treated for control (90% water capacity), and the other 4 replications were treated for drought (15-20% water capacity)	DAP 37 ~ DAP 71	daily intervals

*RAP represents rice automatic phenotyping platform in Huazhong Agricultural University, China

*NPPC represents National Plant Phenomics Centre, IBERS, Aberystwyth University, UK
