# Supplementary material for: Novel Digital Features Discriminate Between Drought Resistant and Drought Sensitive Rice Under Controlled and Field Conditions
Source: Front Plant Sci. 2018 Apr 17;9:492. doi: 10.3389/fpls.2018.00492 (PMC5913589; doi:10.3389/fpls.2018.00492)
Supplement: Supplementary Presentation 4 — The detail of image analysis for rice under field conditions. [file Presentation4.PDF]

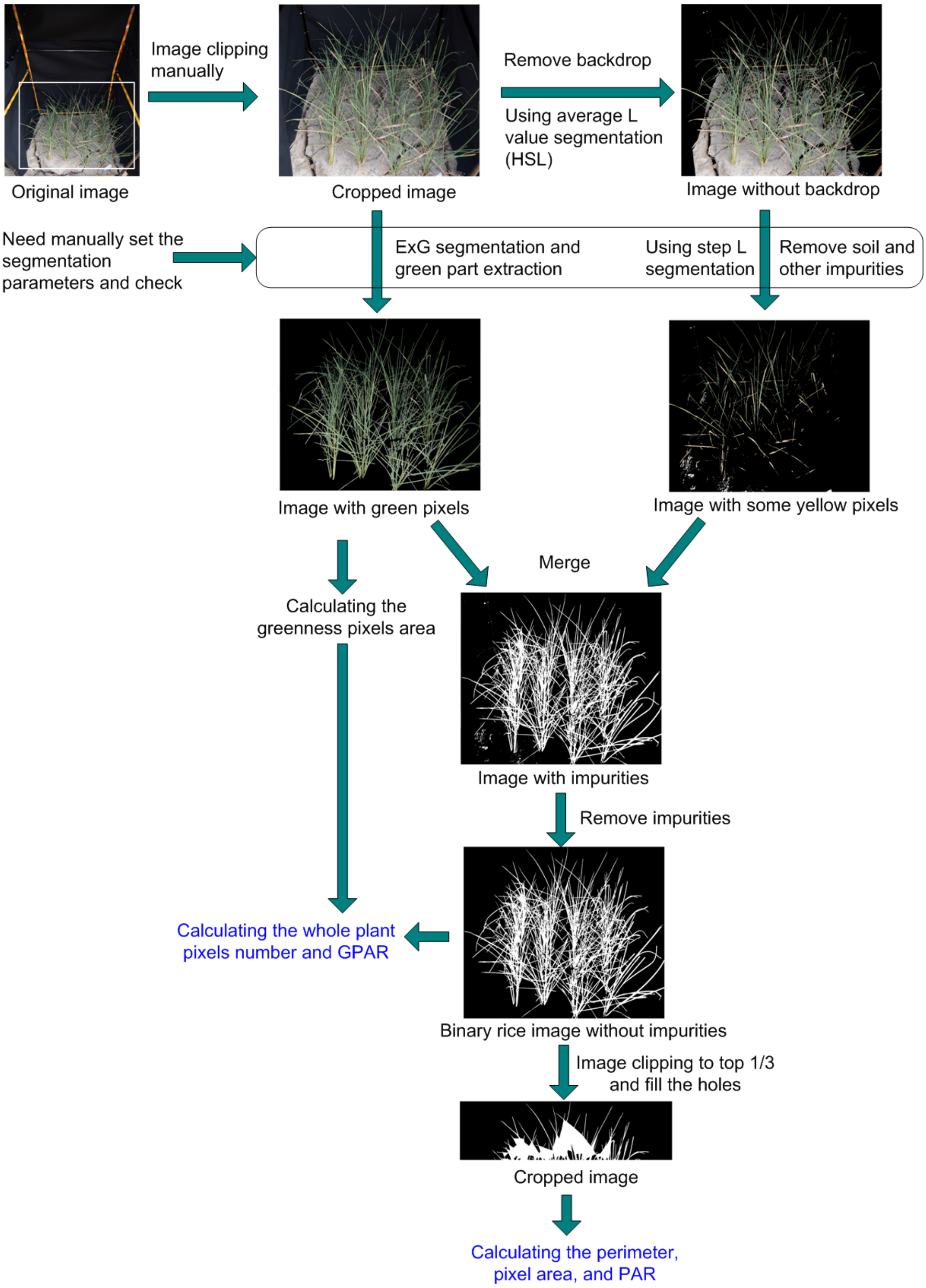

The detail of image analysis for rice under field conditions: (1) After original images were captured in field plot, manually clip the image to select the region of interest; (2) using ExG segmentation algorithm to extract the green part and calculate the green pixels number; (3) using average L value segmentation to remove the backdrop, then

using step L segmentation to remove soil and impurities to obtain the image with yellow pixels; (4) merge the image with green pixels and image with yellow pixels together to obtain the binary image of whole plot with impurities; (5) removing small impurities to get the binary image of whole rice plot without impurities; (6) clip the whole rice plot image to top 1/3 and fill the holes; (7) using the clipped image to calculate the perimeter, pixel number (plant area in clipped image), and PAR.

$$GPAR=\frac{Greenness\ area}{plant\ area}$$

$$PAR=\frac{plant\ perimeter\ in\ clipped\ image}{plant\ area\ in\ clipped\ image}$$
