# Supplementary material for: Novel Digital Features Discriminate Between Drought Resistant and Drought Sensitive Rice Under Controlled and Field Conditions
Source: Front Plant Sci. 2018 Apr 17;9:492. doi: 10.3389/fpls.2018.00492 (PMC5913589; doi:10.3389/fpls.2018.00492)
Supplement: Supplementary Presentation 5 — Results of statistical analysis in this study. [file Presentation5.PDF]

List of Supplementary Presentation 5:

1. **Supplementary Table 1. ANOVA with subsequent post-hoc pairwise comparison using Tukey Honest Significant Difference to test of replication effect in rice under controlled conditions**
2. **Supplementary Table 2. Examination of the ability of the drought-related features in discriminating between drought resistant and drought sensitive accessions in rice under controlled conditions using ANOVA**
3. **Supplementary Table 3. Test of the replication effect in rice under field conditions using ANOVA**
4. **Supplementary Table 4. Examination of the ability of the drought-related features in discriminating between drought resistant and drought sensitive accessions in rice under field conditions using ANOVA**
5. **Supplementary Table 5. ANOVA with subsequent post-hoc pairwise comparison using Tukey Honest Significant Difference to test of replication effect under controlled conditions using the 42 rice accessions in field experiment**
6. **Supplementary Table 6. ANOVA analysis to examine the ability of the drought-related features in discriminating between drought resistant and drought sensitive accessions under controlled conditions using the 42 rice accessions in field experiment**
7. **Supplementary Table 7. Correlation analysis to study the relevance of the drought-related features under controlled conditions and field conditions.**
8. **Supplementary Table 8. ANOVA with subsequent post-hoc pairwise comparison using Tukey Honest Significant Difference to test of replication effect in *Miscanthus***
9. **Supplementary Table 9. Test of the water treatment effect in *Miscanthus* using ANOVA**

Supplementary Table 1. ANOVA with subsequent post-hoc pairwise comparison using Tukey Honest Significant Difference to test the replication effect under controlled conditions

Trait	(I) Replication	(J) Replication	Before stress			After stress		
			Mean Difference (I-J)	Std. Error	Sig.	Mean Difference (I-J)	Std. Error	Sig.
GPAR	1	2	-0.008	0.012	.927	-0.035	0.053	.914
		3	0.001	0.012	1.000	-0.023	0.053	.973
		4	0.008	0.012	.928	-0.040	0.054	.877
	2	1	0.008	0.012	.927	0.035	0.053	.914
		3	0.009	0.012	.895	0.012	0.053	.996
		4	0.015	0.012	.618	-0.006	0.054	1.000
	3	1	-0.001	0.012	1.000	0.023	0.053	.973
		2	-0.009	0.012	.895	-0.012	0.053	.996
		4	0.007	0.012	.953	-0.017	0.054	.988
	4	1	-0.008	0.012	.928	0.040	0.054	.877
		2	-0.015	0.012	.618	0.006	0.054	1.000
		3	-0.007	0.012	.953	0.017	0.054	.988
PAR	1	2	-0.007	0.007	.741	-0.008	0.012	.897
		3	-0.003	0.007	.967	-0.005	0.012	.971
		4	-0.003	0.007	.980	-0.005	0.012	.976
	2	1	0.007	0.007	.741	0.008	0.012	.897
		3	0.004	0.007	.946	0.003	0.012	.994
		4	0.004	0.007	.930	0.003	0.012	.993
	3	1	0.003	0.007	.967	0.005	0.012	.971
		2	-0.004	0.007	.946	-0.003	0.012	.994
		4	0.000	0.007	1.000	0.000	0.012	1.000
	4	1	0.003	0.007	.980	0.005	0.012	.976
		2	-0.004	0.007	.930	-0.003	0.012	.993
		3	0.000	0.007	1.000	0.000	0.012	1.000
TBR	1	2	0.002	0.007	.993	0.001	0.016	1.000
		3	0.005	0.007	.897	0.003	0.016	.998
		4	0.000	0.007	1.000	0.006	0.016	.984
	2	1	-0.002	0.007	.993	-0.001	0.016	1.000
		3	0.003	0.007	.973	0.001	0.016	1.000
		4	-0.001	0.007	.997	0.004	0.016	.993

	3	1	-0.005	0.007	.897	-0.003	0.016	.998
		2	-0.003	0.007	.973	-0.001	0.016	1.000
		4	-0.004	0.007	.927	0.003	0.016	.998
	4	1	0.000	0.007	1.000	-0.006	0.016	.984
		2	0.001	0.007	.997	-0.004	0.016	.993
		3	0.004	0.007	.927	-0.003	0.016	.998
TCR	1	2	0.004	0.008	.964	0.004	0.019	.995
		3	0.003	0.008	.990	0.008	0.019	.973
		4	-0.002	0.008	.996	0.010	0.019	.952
	2	1	-0.004	0.008	.964	-0.004	0.019	.995
		3	-0.001	0.008	.998	0.004	0.019	.997
		4	-0.006	0.008	.904	0.005	0.019	.991
	3	1	-0.003	0.008	.990	-0.008	0.019	.973
		2	0.001	0.008	.998	-0.004	0.019	.997
		4	-0.004	0.008	.955	0.002	0.019	1.000
	4	1	0.002	0.008	.996	-0.010	0.019	.952
		2	0.006	0.008	.904	-0.005	0.019	.991
		3	0.004	0.008	.955	-0.002	0.019	1.000

Supplementary Table 2. Examination of the ability of the drought-related features in discriminating between drought resistant and drought sensitive accessions under controlled conditions using ANOVA

Time point	Trait	DR (mean±std)	DS (mean±std)	Sig.
Before stress	GPAR	0.99±0.041	0.972±0.045	0.189
	PAR	0.194±0.025	0.201±0.028	0.456
	TBR	0.221±0.026	0.224±0.022	0.788
	TCR	0.295±0.032	0.292±0.029	0.758
After stress	GPAR	0.531±0.151	0.14±0.062	4.663E-13
	PAR	0.275±0.035	0.187±0.017	3.078E-12
	TBR	0.187±0.029	0.302±0.033	2.202E-14
	TCR	0.244±0.032	0.383±0.037	2.261E-15

Supplementary Table 3. Test of the replication effect under field conditions using ANOVA

Time point	Trait	Replication 1 (mean±std)	Replication 2 (mean±std)	Sig.
C	GPAR	0.995±0.008	0.995±0.011	.944
	PAR	0.035±0.018	0.033±0.012	.483
D1	GPAR	0.929±0.058	0.893±0.153	.357
	PAR	0.037±0.012	0.034±0.018	.525
D2	GPAR	0.899±0.085	0.819±0.175	.054
	PAR	0.041±0.016	0.044±0.026	.634
D3	GPAR	0.707±0.197	0.659±0.278	.367
	PAR	0.043±0.02	0.051±0.037	.192
Re	GPAR	0.762±0.209	0.768±0.223	.898
	PAR	0.04±0.016	0.034±0.021	.102

Supplementary Table 4. Examination of the ability of the drought-related features in discriminating between drought resistant and drought sensitive accessions under field conditions using ANOVA

Time point	Trait	DR (mean±std)	DS (mean±std)	ANOVA Sig.
C	GPAR	0.996±0.006	0.994±0.007	.317
	PAR	0.034±0.011	0.034±0.016	.855
D1	GPAR	0.957±0.046	0.831±0.129	4.432E-05
	PAR	0.039±0.011	0.022±0.016	1.242E-04
D2	GPAR	0.928±0.035	0.717±0.207	6.006E-04
	PAR	0.05±0.019	0.034±0.016	0.028
D3	GPAR	0.821±0.091	0.399±0.193	5.572E-12
	PAR	0.056±0.02	0.03±0.009	2.617E-05
Re	GPAR	0.853±0.119	0.58±0.244	1.596E-05
	PAR	0.041±0.016	0.03±0.012	0.029

Supplementary Table 5. ANOVA with subsequent post-hoc pairwise comparison using Tukey Honest Significant Difference to test of replication effect under controlled conditions using the 42 rice accessions in field experiment

Trait	(I) Replication	(J) Replication	Before stress			After stress		
			Mean Difference (I-J)	Std. Error	Sig.	Mean Difference (I-J)	Std. Error	Sig.
GPAR	1	2	-0.004	0.010	-0.004	-0.003	0.037	1.000
		3	-0.005	0.010	-0.005	0.007	0.037	0.998
		4	-0.002	0.010	-0.002	0.003	0.037	1.000
	2	1	0.004	0.010	0.004	0.003	0.037	1.000
		3	-0.001	0.010	-0.001	0.009	0.037	0.995
		4	0.002	0.010	0.002	0.006	0.037	0.999
	3	1	0.005	0.010	0.005	-0.007	0.037	0.998
		2	0.001	0.010	0.001	-0.009	0.037	0.995
		4	0.003	0.010	0.003	-0.003	0.037	1.000
	4	1	0.002	0.010	0.002	-0.003	0.037	1.000
		2	-0.002	0.010	-0.002	-0.006	0.037	0.999
		3	-0.003	0.010	-0.003	0.003	0.037	1.000
PAR	1	2	-0.003	0.008	-0.003	-0.005	0.007	0.913
		3	0.002	0.008	0.002	-0.006	0.007	0.827
		4	-0.002	0.008	-0.002	-0.004	0.007	0.963
	2	1	0.003	0.008	0.003	0.005	0.007	0.913
		3	0.005	0.008	0.005	-0.001	0.007	0.997
		4	0.001	0.008	0.001	0.001	0.007	0.998
	3	1	-0.002	0.008	-0.002	0.006	0.007	0.827
		2	-0.005	0.008	-0.005	0.001	0.007	0.997
		4	-0.004	0.008	-0.004	0.003	0.007	0.982
	4	1	0.002	0.008	0.002	0.004	0.007	0.963
		2	-0.001	0.008	-0.001	-0.001	0.007	0.998
		3	0.004	0.008	0.004	-0.003	0.007	0.982
TBR	1	2	-0.001	0.009	-0.001	0.004	0.010	0.973
		3	-0.001	0.009	-0.001	0.003	0.010	0.992
		4	0.004	0.009	0.004	0.010	0.010	0.746
	2	1	0.001	0.009	0.001	-0.004	0.010	0.973
		3	0.000	0.009	0.000	-0.001	0.010	0.999
		4	0.005	0.009	0.005	0.006	0.010	0.940
	3	1	0.001	0.009	0.001	-0.003	0.010	0.992

		2	0.000	0.009	0.000	0.001	0.010	0.999
		4	0.006	0.009	0.006	0.007	0.010	0.890
	4	1	-0.004	0.009	-0.004	-0.010	0.010	0.746
		2	-0.005	0.009	-0.005	-0.006	0.010	0.940
		3	-0.006	0.009	-0.006	-0.007	0.010	0.890
TCR	1	2	0.000	0.011	0.000	0.005	0.012	0.978
		3	0.002	0.011	0.002	0.004	0.012	0.984
		4	0.005	0.011	0.005	0.009	0.012	0.887
	2	1	0.000	0.011	0.000	-0.005	0.012	0.978
		3	0.002	0.011	0.002	-0.001	0.012	1.000
		4	0.005	0.011	0.005	0.004	0.012	0.989
	3	1	-0.002	0.011	-0.002	-0.004	0.012	0.984
		2	-0.002	0.011	-0.002	0.001	0.012	1.000
		4	0.003	0.011	0.003	0.004	0.012	0.983
	4	1	-0.005	0.011	-0.005	-0.009	0.012	0.887
		2	-0.005	0.011	-0.005	-0.004	0.012	0.989
		3	-0.003	0.011	-0.003	-0.004	0.012	0.983

Supplementary Table 6. ANOVA analysis to examine the ability of the drought-related features in discriminating between drought resistant and drought sensitive accessions under controlled conditions using the 42 rice accessions in field experiment

Time point	Trait	DR (mean±std)	DS (mean±std)	ANOVA Sig.
Before stress	GPAR	0.984±0.052	0.993±0.016	0.495
	PAR	0.201±0.037	0.203±0.02	0.882
	TBR	0.223±0.041	0.213±0.031	0.452
	TCR	0.293±0.049	0.285±0.039	0.583
After stress	GPAR	0.391±0.135	0.247±0.118	1.354E-03
	PAR	0.249±0.029	0.221±0.023	2.584E-03
	TBR	0.204±0.033	0.244±0.036	7.806E-04
	TCR	0.266±0.039	0.316±0.043	4.862E-04

Supplementary Table 7. Correlation analysis to study the relevance of the drought-related features under controlled conditions and field conditions. C_GPAR_Field: GPAR change under field conditions. C_GPAR_Control:GPAR change under controlled conditions. C_PAR_Field: PAR change under field

conditions. C_PAR_Control:PAR change under controlled conditions.

		C_GPAR_Field	C_GPAR_Control
C_GPAR_Field	Pearson Correlation	1	.360*
	Sig. (2-tailed)		.019
	N	42	42
C_GPAR_Control	Pearson Correlation	.360*	1
	Sig. (2-tailed)	.019	
	N	42	42
		C_PAR_Field	C_PAR_Control
C_PAR_Field	Pearson Correlation	1	.322*
	Sig. (2-tailed)		.037
	N	42	42
C_PAR_Control	Pearson Correlation	.322*	1
	Sig. (2-tailed)	.037	
	N	42	42

Supplementary Table 8. ANOVA with subsequent post-hoc pairwise comparison using Tukey Honest Significant Difference to test of replication effect in *Miscanthus*

Time point	(I) Replication	(J) Replication	Drought group Mean Difference (I-J)	Drought group Std. Error	Drought group Sig.	Control group Mean Difference (I-J)	Control group Std. Error	Control group Sig.
D1	1	2	0.007	0.031	.995	0.005	0.030	.999
		3	0.019	0.031	.934	-0.003	0.030	1.000
		4	-0.007	0.031	.996	-0.009	0.030	.991
	2	1	-0.007	0.031	.995	-0.005	0.030	.999
		3	0.011	0.031	.984	-0.008	0.030	.994
		4	-0.014	0.031	.967	-0.014	0.030	.969
	3	1	-0.019	0.031	.934	0.003	0.030	1.000
		2	-0.011	0.031	.984	0.008	0.030	.994
		4	-0.025	0.031	.846	-0.006	0.030	.998
	4	1	0.007	0.031	.996	0.009	0.030	.991
		2	0.014	0.031	.967	0.014	0.030	.969
		3	0.025	0.031	.846	0.006	0.030	.998
D2	1	2	0.015	0.032	.967	0.011	0.029	.982
		3	0.017	0.032	.949	0.007	0.030	.995
		4	-0.001	0.032	1.000	-0.001	0.029	1.000
	2	1	-0.015	0.032	.967	-0.011	0.029	.982
		3	0.003	0.032	1.000	-0.004	0.030	.999
		4	-0.016	0.032	.959	-0.012	0.029	.979
	3	1	-0.017	0.032	.949	-0.007	0.030	.995
		2	-0.003	0.032	1.000	0.004	0.030	.999
		4	-0.019	0.032	.939	-0.008	0.030	.994
	4	1	0.001	0.032	1.000	0.001	0.029	1.000
		2	0.016	0.032	.959	0.012	0.029	.979
		3	0.019	0.032	.939	0.008	0.030	.994
D3	1	2	0.003	0.032	1.000	-0.009	0.030	.990
		3	0.008	0.032	.996	-0.011	0.030	.984
		4	-0.006	0.032	.997	-0.003	0.030	1.000
	2	1	-0.003	0.032	1.000	0.009	0.030	.990
		3	0.004	0.032	.999	-0.002	0.030	1.000
		4	-0.010	0.032	.991	0.006	0.030	.997
	3	1	-0.008	0.032	.996	0.011	0.030	.984
		2	-0.004	0.032	.999	0.002	0.030	1.000
		4	-0.014	0.032	.974	0.008	0.030	.994
	4	1	0.006	0.032	.997	0.003	0.030	1.000
		2	0.010	0.032	.991	-0.006	0.030	.997

		3	0.014	0.032	.974	-0.008	0.030	.994
D4	1	2	-0.001	0.033	1.000	-0.013	0.031	.973
		3	-0.015	0.033	.969	-0.007	0.031	.995
		4	-0.018	0.033	.946	0.004	0.031	.999
	2	1	0.001	0.033	1.000	0.013	0.031	.973
		3	-0.014	0.033	.973	0.006	0.031	.997
		4	-0.017	0.033	.953	0.017	0.031	.946
	3	1	0.015	0.033	.969	0.007	0.031	.995
		2	0.014	0.033	.973	-0.006	0.031	.997
		4	-0.003	0.033	1.000	0.011	0.031	.985
	4	1	0.018	0.033	.946	-0.004	0.031	.999
		2	0.017	0.033	.953	-0.017	0.031	.946
		3	0.003	0.033	1.000	-0.011	0.031	.985
D5	1	2	0.002	0.031	1.000	-0.021	0.032	.919
		3	-0.013	0.031	.975	-0.001	0.032	1.000
		4	0.002	0.031	1.000	0.008	0.032	.994
	2	1	-0.002	0.031	1.000	0.021	0.032	.919
		3	-0.015	0.031	.963	0.020	0.032	.928
		4	0.001	0.031	1.000	0.029	0.032	.803
	3	1	0.013	0.031	.975	0.001	0.032	1.000
		2	0.015	0.031	.963	-0.020	0.032	.928
		4	0.015	0.031	.960	0.009	0.032	.992
	4	1	-0.002	0.031	1.000	-0.008	0.032	.994
		2	-0.001	0.031	1.000	-0.029	0.032	.803
		3	-0.015	0.031	.960	-0.009	0.032	.992
D6	1	2	0.000	0.031	1.000	-0.021	0.032	.910
		3	-0.019	0.031	.930	0.002	0.032	1.000
		4	0.005	0.032	.998	0.004	0.032	.999
	2	1	0.000	0.031	1.000	0.021	0.032	.910
		3	-0.019	0.031	.934	0.023	0.032	.885
		4	0.006	0.032	.998	0.025	0.032	.855
	3	1	0.019	0.031	.930	-0.002	0.032	1.000
		2	0.019	0.031	.934	-0.023	0.032	.885
		4	0.024	0.032	.867	0.002	0.032	1.000
	4	1	-0.005	0.032	.998	-0.004	0.032	.999
		2	-0.006	0.032	.998	-0.025	0.032	.855
		3	-0.024	0.032	.867	-0.002	0.032	1.000
D7	1	2	-0.005	0.030	.999	-0.017	0.031	.948
		3	-0.015	0.030	.963	0.007	0.032	.997
		4	-0.002	0.031	1.000	0.000	0.031	1.000
	2	1	0.005	0.030	.999	0.017	0.031	.948
		3	-0.010	0.030	.988	0.024	0.032	.877
		4	0.003	0.031	1.000	0.017	0.031	.947
	3	1	0.015	0.030	.963	-0.007	0.032	.997

		2	0.010	0.030	.988	-0.024	0.032	.877
		4	0.013	0.031	.976	-0.006	0.032	.997
		1	0.002	0.031	1.000	0.000	0.031	1.000
	4	2	-0.003	0.031	1.000	-0.017	0.031	.947
		3	-0.013	0.031	.976	0.006	0.032	.997
D8	1	2	0.001	0.033	1.000	-0.016	0.033	.961
		3	-0.023	0.033	.899	-0.001	0.033	1.000
		4	-0.002	0.033	1.000	0.004	0.033	.999
	2	1	-0.001	0.033	1.000	0.016	0.033	.961
		3	-0.024	0.033	.891	0.015	0.033	.971
		4	-0.003	0.033	1.000	0.020	0.033	.930
	3	1	0.023	0.033	.899	0.001	0.033	1.000
		2	0.024	0.033	.891	-0.015	0.033	.971
		4	0.021	0.033	.927	0.005	0.033	.999
	4	1	0.002	0.033	1.000	-0.004	0.033	.999
		2	0.003	0.033	1.000	-0.020	0.033	.930
		3	-0.021	0.033	.927	-0.005	0.033	.999
D9	1	2	-0.012	0.032	.981	-0.015	0.032	.969
		3	-0.024	0.032	.871	-0.008	0.032	.996
		4	-0.011	0.032	.987	-0.008	0.032	.994
	2	1	0.012	0.032	.981	0.015	0.032	.969
		3	-0.012	0.032	.981	0.007	0.032	.996
		4	0.002	0.032	1.000	0.006	0.032	.997
	3	1	0.024	0.032	.871	0.008	0.032	.996
		2	0.012	0.032	.981	-0.007	0.032	.996
		4	0.014	0.032	.974	-0.001	0.032	1.000
	4	1	0.011	0.032	.987	0.008	0.032	.994
		2	-0.002	0.032	1.000	-0.006	0.032	.997
		3	-0.014	0.032	.974	0.001	0.032	1.000
D10	1	2	-0.004	0.033	1.000	-0.010	0.032	.991
		3	-0.014	0.033	.977	-0.003	0.033	1.000
		4	-0.014	0.033	.973	-0.002	0.032	1.000
	2	1	0.004	0.033	1.000	0.010	0.032	.991
		3	-0.010	0.033	.990	0.007	0.033	.997
		4	-0.011	0.033	.988	0.008	0.032	.995
	3	1	0.014	0.033	.977	0.003	0.033	1.000
		2	0.010	0.033	.990	-0.007	0.033	.997
		4	-0.001	0.033	1.000	0.001	0.033	1.000
	4	1	0.014	0.033	.973	0.002	0.032	1.000
		2	0.011	0.033	.988	-0.008	0.032	.995
		3	0.001	0.033	1.000	-0.001	0.033	1.000
D11	1	2	-0.004	0.033	.999	-0.014	0.031	.969
		3	-0.028	0.033	.833	-0.004	0.032	.999
		4	-0.015	0.033	.968	-0.004	0.031	.999

		1	0.004	0.033	.999	0.014	0.031	.969
	2	3	-0.024	0.033	.884	0.010	0.032	.990
		4	-0.012	0.033	.985	0.010	0.031	.988
		1	0.028	0.033	.833	0.004	0.032	.999
	3	2	0.024	0.033	.884	-0.010	0.032	.990
		4	0.013	0.033	.981	0.001	0.032	1.000
		1	0.015	0.033	.968	0.004	0.031	.999
	4	2	0.012	0.033	.985	-0.010	0.031	.988
		3	-0.013	0.033	.981	-0.001	0.032	1.000
		2	-0.007	0.034	.997	-0.013	0.032	.979
	1	3	-0.039	0.034	.662	-0.006	0.033	.998
		4	-0.020	0.034	.935	-0.009	0.032	.992
		1	0.007	0.034	.997	0.013	0.032	.979
	2	3	-0.032	0.034	.788	0.007	0.033	.997
		4	-0.013	0.034	.982	0.004	0.032	.999
D12		1	0.039	0.034	.662	0.006	0.033	.998
	3	2	0.032	0.034	.788	-0.007	0.033	.997
		4	0.019	0.034	.947	-0.003	0.033	1.000
		1	0.020	0.034	.935	0.009	0.032	.992
	4	2	0.013	0.034	.982	-0.004	0.032	.999
		3	-0.019	0.034	.947	0.003	0.033	1.000
		2	0.011	0.034	.988	-0.011	0.032	.988
	1	3	-0.017	0.034	.963	-0.003	0.032	1.000
		4	-0.003	0.034	1.000	-0.004	0.032	.999
		1	-0.011	0.034	.988	0.011	0.032	.988
	2	3	-0.028	0.034	.851	0.008	0.032	.995
		4	-0.014	0.034	.977	0.007	0.032	.997
D13		1	0.017	0.034	.963	0.003	0.032	1.000
	3	2	0.028	0.034	.851	-0.008	0.032	.995
		4	0.014	0.035	.979	-0.001	0.032	1.000
		1	0.003	0.034	1.000	0.004	0.032	.999
	4	2	0.014	0.034	.977	-0.007	0.032	.997
		3	-0.014	0.035	.979	0.001	0.032	1.000
		2	0.024	0.039	.931	-0.009	0.032	.992
	1	3	-0.003	0.039	1.000	0.000	0.032	1.000
		4	-0.015	0.039	.980	-0.008	0.032	.994
		1	-0.024	0.039	.931	0.009	0.032	.992
	2	3	-0.026	0.039	.911	0.009	0.032	.992
D14		4	-0.039	0.039	.753	0.001	0.032	1.000
		1	0.003	0.039	1.000	0.000	0.032	1.000
	3	2	0.026	0.039	.911	-0.009	0.032	.992
		4	-0.013	0.039	.988	-0.008	0.032	.994
	4	1	0.015	0.039	.980	0.008	0.032	.994
		2	0.039	0.039	.753	-0.001	0.032	1.000

		3	0.013	0.039	.988	0.008	0.032	.994
		2	0.003	0.031	1.000	-0.005	0.033	.999
	1	3	-0.019	0.031	.927	0.005	0.033	.999
		4	-0.014	0.031	.972	-0.011	0.033	.986
		1	-0.003	0.031	1.000	0.005	0.033	.999
	2	3	-0.022	0.031	.888	0.010	0.033	.991
		4	-0.017	0.031	.948	-0.006	0.033	.998
D15		1	0.019	0.031	.927	-0.005	0.033	.999
	3	2	0.022	0.031	.888	-0.010	0.033	.991
		4	0.005	0.031	.998	-0.016	0.033	.962
		1	0.014	0.031	.972	0.011	0.033	.986
	4	2	0.017	0.031	.948	0.006	0.033	.998
		3	-0.005	0.031	.998	0.016	0.033	.962
		2	-0.003	0.031	1.000	-0.002	0.032	1.000
	1	3	-0.022	0.031	.896	0.004	0.033	1.000
		4	-0.014	0.031	.970	-0.015	0.032	.968
		1	0.003	0.031	1.000	0.002	0.032	1.000
	2	3	-0.019	0.031	.925	0.005	0.033	.998
		4	-0.012	0.031	.983	-0.013	0.032	.978
D16		1	0.022	0.031	.896	-0.004	0.033	1.000
	3	2	0.019	0.031	.925	-0.005	0.033	.998
		4	0.008	0.031	.994	-0.018	0.033	.942
		1	0.014	0.031	.970	0.015	0.032	.968
	4	2	0.012	0.031	.983	0.013	0.032	.978
		3	-0.008	0.031	.994	0.018	0.033	.942
		2	-0.012	0.033	.983	-0.001	0.033	1.000
	1	3	-0.034	0.033	.735	0.007	0.033	.997
		4	-0.021	0.033	.926	-0.016	0.033	.960
		1	0.012	0.033	.983	0.001	0.033	1.000
	2	3	-0.022	0.033	.912	0.008	0.033	.995
		4	-0.008	0.033	.994	-0.015	0.033	.967
D17		1	0.034	0.033	.735	-0.007	0.033	.997
	3	2	0.022	0.033	.912	-0.008	0.033	.995
		4	0.014	0.033	.977	-0.023	0.033	.896
		1	0.021	0.033	.926	0.016	0.033	.960
	4	2	0.008	0.033	.994	0.015	0.033	.967
		3	-0.014	0.033	.977	0.023	0.033	.896
		2	-0.017	0.035	.962	-0.003	0.034	1.000
	1	3	-0.043	0.035	.611	0.005	0.034	.999
		4	-0.014	0.035	.978	-0.012	0.034	.983
D18		1	0.017	0.035	.962	0.003	0.034	1.000
	2	3	-0.026	0.035	.878	0.008	0.034	.995
		4	0.003	0.035	1.000	-0.009	0.034	.992
	3	1	0.043	0.035	.611	-0.005	0.034	.999

		2	0.026	0.035	.878	-0.008	0.034	.995
		4	0.029	0.035	.841	-0.018	0.034	.955
		1	0.014	0.035	.978	0.012	0.034	.983
	4	2	-0.003	0.035	1.000	0.009	0.034	.992
		3	-0.029	0.035	.841	0.018	0.034	.955
		2	-0.017	0.034	.959	-0.004	0.033	1.000
	1	3	-0.039	0.034	.668	-0.002	0.033	1.000
		4	0.005	0.034	.999	-0.016	0.033	.960
		1	0.017	0.034	.959	0.004	0.033	1.000
	2	3	-0.022	0.034	.918	0.001	0.033	1.000
		4	0.022	0.034	.920	-0.013	0.033	.980
D19		1	0.039	0.034	.668	0.002	0.033	1.000
	3	2	0.022	0.034	.918	-0.001	0.033	1.000
		4	0.044	0.034	.586	-0.014	0.033	.974
		1	-0.005	0.034	.999	0.016	0.033	.960
	4	2	-0.022	0.034	.920	0.013	0.033	.980
		3	-0.044	0.034	.586	0.014	0.033	.974
		2	-0.004	0.036	.999	0.000	0.034	1.000
	1	3	-0.038	0.036	.711	0.005	0.034	.999
		4	0.006	0.036	.998	-0.011	0.034	.989
		1	0.004	0.036	.999	0.000	0.034	1.000
	2	3	-0.034	0.036	.773	0.005	0.034	.999
		4	0.010	0.036	.992	-0.011	0.034	.987
D20		1	0.038	0.036	.711	-0.005	0.034	.999
	3	2	0.034	0.036	.773	-0.005	0.034	.999
		4	0.045	0.036	.610	-0.016	0.034	.966
		1	-0.006	0.036	.998	0.011	0.034	.989
	4	2	-0.010	0.036	.992	0.011	0.034	.987
		3	-0.045	0.036	.610	0.016	0.034	.966
		2	0.008	0.035	.996	0.004	0.033	1.000
	1	3	-0.026	0.035	.875	0.006	0.034	.998
		4	0.012	0.035	.986	-0.013	0.033	.980
		1	-0.008	0.035	.996	-0.004	0.033	1.000
	2	3	-0.034	0.035	.764	0.002	0.034	1.000
		4	0.005	0.035	.999	-0.017	0.033	.960
D21		1	0.026	0.035	.875	-0.006	0.034	.998
	3	2	0.034	0.035	.764	-0.002	0.034	1.000
		4	0.039	0.035	.696	-0.019	0.034	.943
		1	-0.012	0.035	.986	0.013	0.033	.980
	4	2	-0.005	0.035	.999	0.017	0.033	.960
		3	-0.039	0.035	.696	0.019	0.034	.943
		2	0.016	0.036	.970	0.003	0.034	1.000
D22	1	3	-0.024	0.036	.915	0.007	0.034	.997
		4	0.017	0.037	.967	-0.014	0.034	.976

	2	1	-0.016	0.036	.970	-0.003	0.034	1.000
		3	-0.040	0.036	.691	0.004	0.034	.999
		4	0.001	0.036	1.000	-0.017	0.034	.961
	3	1	0.024	0.036	.915	-0.007	0.034	.997
		2	0.040	0.036	.691	-0.004	0.034	.999
		4	0.041	0.037	.684	-0.021	0.034	.928
	4	1	-0.017	0.037	.967	0.014	0.034	.976
		2	-0.001	0.036	1.000	0.017	0.034	.961
		3	-0.041	0.037	.684	0.021	0.034	.928
D23	1	2	0.019	0.036	.952	0.001	0.033	1.000
		3	-0.019	0.037	.958	0.005	0.034	.999
		4	0.022	0.037	.936	-0.013	0.033	.979
	2	1	-0.019	0.036	.952	-0.001	0.033	1.000
		3	-0.038	0.036	.728	0.004	0.034	.999
		4	0.002	0.037	1.000	-0.014	0.033	.973
	3	1	0.019	0.037	.958	-0.005	0.034	.999
		2	0.038	0.036	.728	-0.004	0.034	.999
		4	0.040	0.037	.698	-0.018	0.034	.946
	4	1	-0.022	0.037	.936	0.013	0.033	.979
		2	-0.002	0.037	1.000	0.014	0.033	.973
		3	-0.040	0.037	.698	0.018	0.034	.946
D24	1	2	0.013	0.033	.978	0.003	0.034	1.000
		3	-0.035	0.033	.712	0.009	0.034	.994
		4	0.017	0.033	.958	-0.011	0.034	.988
	2	1	-0.013	0.033	.978	-0.003	0.034	1.000
		3	-0.048	0.033	.457	0.006	0.034	.998
		4	0.004	0.033	1.000	-0.014	0.034	.977
	3	1	0.035	0.033	.712	-0.009	0.034	.994
		2	0.048	0.033	.457	-0.006	0.034	.998
		4	0.052	0.033	.405	-0.020	0.034	.938
	4	1	-0.017	0.033	.958	0.011	0.034	.988
		2	-0.004	0.033	1.000	0.014	0.034	.977
		3	-0.052	0.033	.405	0.020	0.034	.938
D25	1	2	0.010	0.037	.993	0.001	0.033	1.000
		3	-0.037	0.037	.744	0.007	0.034	.997
		4	-0.005	0.037	.999	-0.004	0.033	.999
	2	1	-0.010	0.037	.993	-0.001	0.033	1.000
		3	-0.047	0.037	.573	0.006	0.034	.998
		4	-0.015	0.037	.978	-0.005	0.033	.999
	3	1	0.037	0.037	.744	-0.007	0.034	.997
		2	0.047	0.037	.573	-0.006	0.034	.998
		4	0.032	0.037	.815	-0.011	0.034	.988
	4	1	0.005	0.037	.999	0.004	0.033	.999
		2	0.015	0.037	.978	0.005	0.033	.999

		3	-0.032	0.037	.815	0.011	0.034	.988
		2	0.006	0.037	.999	0.005	0.034	.999
	1	3	-0.036	0.038	.772	0.007	0.034	.997
		4	-0.012	0.038	.988	-0.004	0.034	1.000
		1	-0.006	0.037	.999	-0.005	0.034	.999
	2	3	-0.042	0.037	.674	0.002	0.034	1.000
		4	-0.018	0.037	.962	-0.008	0.034	.995
D26		1	0.036	0.038	.772	-0.007	0.034	.997
	3	2	0.042	0.037	.674	-0.002	0.034	1.000
		4	0.024	0.038	.920	-0.011	0.034	.989
		1	0.012	0.038	.988	0.004	0.034	1.000
	4	2	0.018	0.037	.962	0.008	0.034	.995
		3	-0.024	0.038	.920	0.011	0.034	.989
		2	0.012	0.032	.984	-0.001	0.030	1.000
	1	3	-0.016	0.032	.962	-0.006	0.030	.997
		4	0.002	0.033	1.000	-0.003	0.030	1.000
		1	-0.012	0.032	.984	0.001	0.030	1.000
	2	3	-0.027	0.032	.834	-0.005	0.030	.999
		4	-0.010	0.033	.991	-0.001	0.030	1.000
D27		1	0.016	0.032	.962	0.006	0.030	.997
	3	2	0.027	0.032	.834	0.005	0.030	.999
		4	0.018	0.033	.948	0.003	0.030	1.000
		1	-0.002	0.033	1.000	0.003	0.030	1.000
	4	2	0.010	0.033	.991	0.001	0.030	1.000
		3	-0.018	0.033	.948	-0.003	0.030	1.000
		2	0.013	0.037	.986	-0.002	0.034	1.000
	1	3	-0.026	0.037	.895	0.001	0.034	1.000
		4	-0.007	0.037	.998	-0.003	0.034	1.000
		1	-0.013	0.037	.986	0.002	0.034	1.000
	2	3	-0.039	0.037	.716	0.003	0.034	1.000
		4	-0.019	0.037	.953	-0.001	0.034	1.000
D28		1	0.026	0.037	.895	-0.001	0.034	1.000
	3	2	0.039	0.037	.716	-0.003	0.034	1.000
		4	0.020	0.037	.952	-0.005	0.034	.999
		1	0.007	0.037	.998	0.003	0.034	1.000
	4	2	0.019	0.037	.953	0.001	0.034	1.000
		3	-0.020	0.037	.952	0.005	0.034	.999
		2	0.014	0.037	.982	-0.002	0.035	1.000
	1	3	-0.022	0.037	.936	0.006	0.035	.998
		4	-0.009	0.037	.996	0.000	0.035	1.000
D29		1	-0.014	0.037	.982	0.002	0.035	1.000
	2	3	-0.035	0.037	.769	0.008	0.035	.996
		4	-0.022	0.037	.930	0.002	0.035	1.000
	3	1	0.022	0.037	.936	-0.006	0.035	.998

		2	0.035	0.037	.769	-0.008	0.035	.996
		4	0.013	0.037	.984	-0.006	0.035	.999
		1	0.009	0.037	.996	0.000	0.035	1.000
	4	2	0.022	0.037	.930	-0.002	0.035	1.000
		3	-0.013	0.037	.984	0.006	0.035	.999
		2	0.001	0.038	1.000	-0.006	0.034	.998
	1	3	-0.028	0.039	.884	0.002	0.034	1.000
		4	-0.020	0.039	.958	-0.006	0.034	.998
		1	-0.001	0.038	1.000	0.006	0.034	.998
	2	3	-0.029	0.038	.873	0.008	0.034	.996
		4	-0.020	0.038	.952	0.000	0.034	1.000
D30		1	0.028	0.039	.884	-0.002	0.034	1.000
	3	2	0.029	0.038	.873	-0.008	0.034	.996
		4	0.009	0.039	.996	-0.008	0.034	.996
		1	0.020	0.039	.958	0.006	0.034	.998
	4	2	0.020	0.038	.952	0.000	0.034	1.000
		3	-0.009	0.039	.996	0.008	0.034	.996
		2	0.004	0.038	1.000	0.000	0.034	1.000
	1	3	-0.030	0.039	.871	0.005	0.034	.999
		4	-0.018	0.039	.965	-0.001	0.034	1.000
		1	-0.004	0.038	1.000	0.000	0.034	1.000
	2	3	-0.034	0.038	.817	0.005	0.034	.999
		4	-0.022	0.038	.937	-0.001	0.034	1.000
D31		1	0.030	0.039	.871	-0.005	0.034	.999
	3	2	0.034	0.038	.817	-0.005	0.034	.999
		4	0.011	0.039	.991	-0.006	0.034	.998
		1	0.018	0.039	.965	0.001	0.034	1.000
	4	2	0.022	0.038	.937	0.001	0.034	1.000
		3	-0.011	0.039	.991	0.006	0.034	.998
		2	0.008	0.038	.996	0.001	0.036	1.000
	1	3	-0.029	0.038	.871	0.010	0.036	.991
		4	-0.006	0.038	.998	0.001	0.035	1.000
		1	-0.008	0.038	.996	-0.001	0.036	1.000
	2	3	-0.038	0.038	.754	0.009	0.036	.994
		4	-0.015	0.038	.980	0.000	0.036	1.000
D32		1	0.029	0.038	.871	-0.010	0.036	.991
	3	2	0.038	0.038	.754	-0.009	0.036	.994
		4	0.023	0.038	.932	-0.009	0.036	.994
		1	0.006	0.038	.998	-0.001	0.035	1.000
	4	2	0.015	0.038	.980	0.000	0.036	1.000
		3	-0.023	0.038	.932	0.009	0.036	.994
		2	0.010	0.038	.993	-0.008	0.034	.996
D33	1	3	-0.030	0.039	.862	0.002	0.034	1.000
		4	0.007	0.039	.998	-0.001	0.034	1.000

		1	-0.010	0.038	.993	0.008	0.034	.996
	2	3	-0.041	0.038	.716	0.010	0.034	.991
		4	-0.003	0.038	1.000	0.006	0.034	.998
		1	0.030	0.039	.862	-0.002	0.034	1.000
	3	2	0.041	0.038	.716	-0.010	0.034	.991
		4	0.037	0.039	.771	-0.004	0.034	1.000
		1	-0.007	0.039	.998	0.001	0.034	1.000
	4	2	0.003	0.038	1.000	-0.006	0.034	.998
		3	-0.037	0.039	.771	0.004	0.034	1.000
		2	0.011	0.039	.993	-0.005	0.035	.999
	1	3	-0.027	0.040	.902	0.005	0.035	.999
		4	0.008	0.040	.997	0.001	0.035	1.000
		1	-0.011	0.039	.993	0.005	0.035	.999
	2	3	-0.038	0.039	.768	0.010	0.035	.993
		4	-0.003	0.039	1.000	0.006	0.035	.998
D34		1	0.027	0.040	.902	-0.005	0.035	.999
	3	2	0.038	0.039	.768	-0.010	0.035	.993
		4	0.035	0.040	.812	-0.003	0.035	1.000
		1	-0.008	0.040	.997	-0.001	0.035	1.000
	4	2	0.003	0.039	1.000	-0.006	0.035	.998
		3	-0.035	0.040	.812	0.003	0.035	1.000
		2	0.012	0.040	.990	-0.004	0.035	.999
	1	3	-0.024	0.040	.928	0.008	0.035	.995
		4	0.010	0.040	.994	0.004	0.035	.999
		1	-0.012	0.040	.990	0.004	0.035	.999
	2	3	-0.036	0.040	.795	0.012	0.035	.985
		4	-0.002	0.040	1.000	0.008	0.035	.995
D35		1	0.024	0.040	.928	-0.008	0.035	.995
	3	2	0.036	0.040	.795	-0.012	0.035	.985
		4	0.035	0.040	.821	-0.004	0.035	.999
		1	-0.010	0.040	.994	-0.004	0.035	.999
	4	2	0.002	0.040	1.000	-0.008	0.035	.995
		3	-0.035	0.040	.821	0.004	0.035	.999

Supplementary Table 9. Test of the drought treatment effect in *Miscanthus* using ANOVA

Time point	Drought group (mean±std)	Control group (mean±std)	Sig.
D1	0.501±0.123	0.503±0.119	.938
D2	0.504±0.125	0.504±0.119	.998
D3	0.501±0.125	0.505±0.12	.883
D4	0.5±0.129	0.493±0.124	.800
D5	0.498±0.125	0.496±0.129	.955
D6	0.503±0.124	0.5±0.129	.913
D7	0.497±0.121	0.496±0.128	.965
D8	0.499±0.13	0.495±0.132	.886
D9	0.494±0.125	0.494±0.131	.997
D10	0.496±0.132	0.491±0.131	.880
D11	0.494±0.131	0.489±0.127	.856
D12	0.495±0.136	0.489±0.131	.856
D13	0.486±0.136	0.476±0.13	.726
D14	0.489±0.148	0.474±0.13	.625
D15	0.483±0.124	0.474±0.132	.754
D16	0.488±0.127	0.47±0.13	.537
D17	0.496±0.132	0.467±0.131	.336
D18	0.501±0.136	0.462±0.135	.210
D19	0.505±0.135	0.46±0.133	.141
D20	0.514±0.14	0.453±0.135	.051
D21	0.523±0.137	0.45±0.131	.019
D22	0.535±0.14	0.447±0.134	.006
D23	0.543±0.142	0.444±0.132	.002
D24	0.528±0.124	0.439±0.135	.003
D25	0.53±0.137	0.423±0.131	7.042E-04
D26	0.534±0.14	0.423±0.132	5.676E-04
D27	0.539±0.127	0.419±0.119	4.671E-05
D28	0.552±0.14	0.418±0.131	3.994E-05
D29	0.546±0.137	0.416±0.135	6.599E-05
D30	0.541±0.142	0.413±0.132	9.413E-05
D31	0.551±0.143	0.413±0.132	2.941E-05
D32	0.546±0.138	0.407±0.136	2.461E-05
D33	0.559±0.138	0.406±0.13	3.066E-06
D34	0.559±0.143	0.405±0.133	4.806E-06
D35	0.553±0.144	0.403±0.133	8.191E-06
