# Supplementary material for: Novel Digital Features Discriminate Between Drought Resistant and Drought Sensitive Rice Under Controlled and Field Conditions
Source: Front Plant Sci. 2018 Apr 17;9:492. doi: 10.3389/fpls.2018.00492 (PMC5913589; doi:10.3389/fpls.2018.00492)
Supplement: Supplementary Presentation 6 — The correlation analysis between controlled conditions and field conditions for GPAR change (10 repeated analysis). [file Presentation6.PDF]

```
CORRELATIONS
  /VARIABLES=VAR00001 VAR00002
  /PRINT=TWOTAIL NOSIG
  /MISSING=PAIRWISE.
```

Correlations

Notes

Output Created		13-MAR-2018 07:04:21
Comments		
Input	Active Dataset	0
	Filter	<none>
	Weight	<none>
	Split File	<none>
	N of Rows in Working Data File	40
Missing Value Handling	Definition of Missing	User-defined missing values are treated as missing.
	Cases Used	Statistics for each pair of variables are based on all the cases with valid data for that pair.
Syntax		CORRELATIONS /VARIABLES=VAR00001 VAR00002 /PRINT=TWOTAIL NOSIG...
Resources	Processor Time	00:00:00.02
	Elapsed Time	00:00:00.00

[0]

Correlations

		VAR00001	VAR00002
VAR00001	Pearson Correlation	1	.349*
	Sig. (2-tailed)		.027
	N	40	40
VAR00002	Pearson Correlation	.349*	1
	Sig. (2-tailed)	.027	
	N	40	40

*. Correlation is significant at the 0.05 level (2-tailed).


```
CORRELATIONS
  /VARIABLES=VAR00001 VAR00002
  /PRINT=TWOTAIL NOSIG
  /MISSING=PAIRWISE.
```

Correlations

Notes

Output Created		13-MAR-2018 07:04:32
Comments		
Input	Active Dataset	0
	Filter	<none>
	Weight	<none>
	Split File	<none>
	N of Rows in Working Data File	40
Missing Value Handling	Definition of Missing	User-defined missing values are treated as missing.
	Cases Used	Statistics for each pair of variables are based on all the cases with valid data for that pair.
Syntax		CORRELATIONS /VARIABLES=VAR00001 VAR00002 /PRINT=TWOTAIL NOSIG...
Resources	Processor Time	00:00:00.00
	Elapsed Time	00:00:00.01

[0]

Correlations

		VAR00001	VAR00002
VAR00001	Pearson Correlation	1	.414**
	Sig. (2-tailed)		.008
	N	40	40
VAR00002	Pearson Correlation	.414**	1
	Sig. (2-tailed)	.008	
	N	40	40

**. Correlation is significant at the 0.01 level (2-tailed).

```
CORRELATIONS
  /VARIABLES=VAR00001 VAR00002
  /PRINT=TWOTAIL NOSIG
  /MISSING=PAIRWISE.
```

Correlations

Notes

Output Created		13-MAR-2018 07:04:44
Comments		
Input	Active Dataset	0
	Filter	<none>
	Weight	<none>
	Split File	<none>
	N of Rows in Working Data File	40
Missing Value Handling	Definition of Missing	User-defined missing values are treated as missing.
	Cases Used	Statistics for each pair of variables are based on all the cases with valid data for that pair.
Syntax		CORRELATIONS /VARIABLES=VAR00001 VAR00002 /PRINT=TWOTAIL NOSIG...
Resources	Processor Time	00:00:00.03
	Elapsed Time	00:00:00.01

[0]

Correlations

		VAR00001	VAR00002
VAR00001	Pearson Correlation	1	.375*
	Sig. (2-tailed)		.017
	N	40	40
VAR00002	Pearson Correlation	.375*	1
	Sig. (2-tailed)	.017	
	N	40	40

*. Correlation is significant at the 0.05 level (2-tailed).

```
CORRELATIONS
  /VARIABLES=VAR00001 VAR00002
  /PRINT=TWOTAIL NOSIG
  /MISSING=PAIRWISE.
```

Correlations

Notes

Output Created		13-MAR-2018 07:04:56
Comments		
Input	Active Dataset	0
	Filter	<none>
	Weight	<none>
	Split File	<none>
	N of Rows in Working Data File	40
Missing Value Handling	Definition of Missing	User-defined missing values are treated as missing.
	Cases Used	Statistics for each pair of variables are based on all the cases with valid data for that pair.
Syntax		CORRELATIONS /VARIABLES=VAR00001 VAR00002 /PRINT=TWOTAIL NOSIG...
Resources	Processor Time	00:00:00.00
	Elapsed Time	00:00:00.02

[0]

Correlations

		VAR00001	VAR00002
VAR00001	Pearson Correlation	1	.369*
	Sig. (2-tailed)		.019
	N	40	40
VAR00002	Pearson Correlation	.369*	1
	Sig. (2-tailed)	.019	
	N	40	40

*. Correlation is significant at the 0.05 level (2-tailed).

```
CORRELATIONS
  /VARIABLES=VAR00001 VAR00002
  /PRINT=TWOTAIL NOSIG
  /MISSING=PAIRWISE.
```

Correlations

Notes

Output Created		13-MAR-2018 07:05:11
Comments		
Input	Active Dataset	0
	Filter	<none>
	Weight	<none>
	Split File	<none>
	N of Rows in Working Data File	40
Missing Value Handling	Definition of Missing	User-defined missing values are treated as missing.
	Cases Used	Statistics for each pair of variables are based on all the cases with valid data for that pair.
Syntax		CORRELATIONS /VARIABLES=VAR00001 VAR00002 /PRINT=TWOTAIL NOSIG...
Resources	Processor Time	00:00:00.00
	Elapsed Time	00:00:00.01

[0]

Correlations

		VAR00001	VAR00002
VAR00001	Pearson Correlation	1	.380*
	Sig. (2-tailed)		.015
	N	40	40
VAR00002	Pearson Correlation	.380*	1
	Sig. (2-tailed)	.015	
	N	40	40

*. Correlation is significant at the 0.05 level (2-tailed).

```
CORRELATIONS
  /VARIABLES=VAR00001 VAR00002
  /PRINT=TWOTAIL NOSIG
  /MISSING=PAIRWISE.
```

Correlations

Notes

Output Created		13-MAR-2018 07:05:27
Comments		
Input	Active Dataset	0
	Filter	<none>
	Weight	<none>
	Split File	<none>
	N of Rows in Working Data File	40
Missing Value Handling	Definition of Missing	User-defined missing values are treated as missing.
	Cases Used	Statistics for each pair of variables are based on all the cases with valid data for that pair.
Syntax		CORRELATIONS /VARIABLES=VAR00001 VAR00002 /PRINT=TWOTAIL NOSIG...
Resources	Processor Time	00:00:00.00
	Elapsed Time	00:00:00.00

[0]

Correlations

		VAR00001	VAR00002
VAR00001	Pearson Correlation	1	.333*
	Sig. (2-tailed)		.036
	N	40	40
VAR00002	Pearson Correlation	.333*	1
	Sig. (2-tailed)	.036	
	N	40	40

*. Correlation is significant at the 0.05 level (2-tailed).

```
CORRELATIONS
  /VARIABLES=VAR00001 VAR00002
  /PRINT=TWOTAIL NOSIG
  /MISSING=PAIRWISE.
```

Correlations

Notes

Output Created		13-MAR-2018 07:05:44
Comments		
Input	Active Dataset	0
	Filter	<none>
	Weight	<none>
	Split File	<none>
	N of Rows in Working Data File	40
Missing Value Handling	Definition of Missing	User-defined missing values are treated as missing.
	Cases Used	Statistics for each pair of variables are based on all the cases with valid data for that pair.
Syntax		CORRELATIONS /VARIABLES=VAR00001 VAR00002 /PRINT=TWOTAIL NOSIG...
Resources	Processor Time	00:00:00.00
	Elapsed Time	00:00:00.01

[0]

Correlations

		VAR00001	VAR00002
VAR00001	Pearson Correlation	1	.362*
	Sig. (2-tailed)		.022
	N	40	40
VAR00002	Pearson Correlation	.362*	1
	Sig. (2-tailed)	.022	
	N	40	40

*. Correlation is significant at the 0.05 level (2-tailed).

```
CORRELATIONS
  /VARIABLES=VAR00001 VAR00002
  /PRINT=TWOTAIL NOSIG
  /MISSING=PAIRWISE.
```

Correlations

Notes

Output Created		13-MAR-2018 07:05:55
Comments		
Input	Active Dataset	0
	Filter	<none>
	Weight	<none>
	Split File	<none>
	N of Rows in Working Data File	40
Missing Value Handling	Definition of Missing	User-defined missing values are treated as missing.
	Cases Used	Statistics for each pair of variables are based on all the cases with valid data for that pair.
Syntax		CORRELATIONS /VARIABLES=VAR00001 VAR00002 /PRINT=TWOTAIL NOSIG...
Resources	Processor Time	00:00:00.02
	Elapsed Time	00:00:00.01

[0]

Correlations

		VAR00001	VAR00002
VAR00001	Pearson Correlation	1	.368*
	Sig. (2-tailed)		.019
	N	40	40
VAR00002	Pearson Correlation	.368*	1
	Sig. (2-tailed)	.019	
	N	40	40

*. Correlation is significant at the 0.05 level (2-tailed).

```
CORRELATIONS
  /VARIABLES=VAR00001 VAR00002
  /PRINT=TWOTAIL NOSIG
  /MISSING=PAIRWISE.
```

Correlations

Notes

Output Created		13-MAR-2018 07:06:15
Comments		
Input	Active Dataset	0
	Filter	<none>
	Weight	<none>
	Split File	<none>
	N of Rows in Working Data File	40
Missing Value Handling	Definition of Missing	User-defined missing values are treated as missing.
	Cases Used	Statistics for each pair of variables are based on all the cases with valid data for that pair.
Syntax		CORRELATIONS /VARIABLES=VAR00001 VAR00002 /PRINT=TWOTAIL NOSIG...
Resources	Processor Time	00:00:00.02
	Elapsed Time	00:00:00.01

[0]

Correlations

		VAR00001	VAR00002
VAR00001	Pearson Correlation	1	.339*
	Sig. (2-tailed)		.033
	N	40	40
VAR00002	Pearson Correlation	.339*	1
	Sig. (2-tailed)	.033	
	N	40	40

*. Correlation is significant at the 0.05 level (2-tailed).

```
CORRELATIONS
  /VARIABLES=VAR00001 VAR00002
  /PRINT=TWOTAIL NOSIG
  /MISSING=PAIRWISE.
```

Correlations

Notes

Output Created		13-MAR-2018 07:06:31
Comments		
Input	Active Dataset	0
	Filter	<none>
	Weight	<none>
	Split File	<none>
	N of Rows in Working Data File	40
Missing Value Handling	Definition of Missing	User-defined missing values are treated as missing.
	Cases Used	Statistics for each pair of variables are based on all the cases with valid data for that pair.
Syntax		CORRELATIONS /VARIABLES=VAR00001 VAR00002 /PRINT=TWOTAIL NOSIG...
Resources	Processor Time	00:00:00.00
	Elapsed Time	00:00:00.01

[0]

Correlations

		VAR00001	VAR00002
VAR00001	Pearson Correlation	1	.411**
	Sig. (2-tailed)		.008
	N	40	40
VAR00002	Pearson Correlation	.411**	1
	Sig. (2-tailed)	.008	
	N	40	40

**. Correlation is significant at the 0.01 level (2-tailed).
