# Supplementary material for: Novel Digital Features Discriminate Between Drought Resistant and Drought Sensitive Rice Under Controlled and Field Conditions
Source: Front Plant Sci. 2018 Apr 17;9:492. doi: 10.3389/fpls.2018.00492 (PMC5913589; doi:10.3389/fpls.2018.00492)
Supplement: Supplementary Presentation 7 — The correlation analysis between controlled conditions and field conditions for PAR change (10 repeated analysis). [file Presentation7.PDF]

```
CORRELATIONS
  /VARIABLES=VAR00001 VAR00002
  /PRINT=TWOTAIL NOSIG
  /MISSING=PAIRWISE.
```

Correlations

Notes

Output Created		13-MAR-2018 06:52:14
Comments		
Input	Active Dataset	0
	Filter	<none>
	Weight	<none>
	Split File	<none>
	N of Rows in Working Data File	40
Missing Value Handling	Definition of Missing	User-defined missing values are treated as missing.
	Cases Used	Statistics for each pair of variables are based on all the cases with valid data for that pair.
Syntax		CORRELATIONS /VARIABLES=VAR00001 VAR00002 /PRINT=TWOTAIL NOSIG...
Resources	Processor Time	00:00:00.00
	Elapsed Time	00:00:00.01

[0]

Correlations

		VAR00001	VAR00002
VAR00001	Pearson Correlation	1	.315*
	Sig. (2-tailed)		.048
	N	40	40
VAR00002	Pearson Correlation	.315*	1
	Sig. (2-tailed)	.048	
	N	40	40

*. Correlation is significant at the 0.05 level (2-tailed).


```
CORRELATIONS
  /VARIABLES=VAR00001 VAR00002
  /PRINT=TWOTAIL NOSIG
  /MISSING=PAIRWISE.
```

Correlations

Notes

Output Created		13-MAR-2018 06:52:31
Comments		
Input	Active Dataset	0
	Filter	<none>
	Weight	<none>
	Split File	<none>
	N of Rows in Working Data File	40
Missing Value Handling	Definition of Missing	User-defined missing values are treated as missing.
	Cases Used	Statistics for each pair of variables are based on all the cases with valid data for that pair.
Syntax		CORRELATIONS /VARIABLES=VAR00001 VAR00002 /PRINT=TWOTAIL NOSIG...
Resources	Processor Time	00:00:00.00
	Elapsed Time	00:00:00.01

[0]

Correlations

		VAR00001	VAR00002
VAR00001	Pearson Correlation	1	.359*
	Sig. (2-tailed)		.023
	N	40	40
VAR00002	Pearson Correlation	.359*	1
	Sig. (2-tailed)	.023	
	N	40	40

*. Correlation is significant at the 0.05 level (2-tailed).

```
CORRELATIONS
  /VARIABLES=VAR00001 VAR00002
  /PRINT=TWOTAIL NOSIG
  /MISSING=PAIRWISE.
```

Correlations

Notes

Output Created		13-MAR-2018 06:53:59
Comments		
Input	Active Dataset	0
	Filter	<none>
	Weight	<none>
	Split File	<none>
	N of Rows in Working Data File	40
Missing Value Handling	Definition of Missing	User-defined missing values are treated as missing.
	Cases Used	Statistics for each pair of variables are based on all the cases with valid data for that pair.
Syntax		CORRELATIONS /VARIABLES=VAR00001 VAR00002 /PRINT=TWOTAIL NOSIG...
Resources	Processor Time	00:00:00.02
	Elapsed Time	00:00:00.01

[0]

Correlations

		VAR00001	VAR00002
VAR00001	Pearson Correlation	1	.361*
	Sig. (2-tailed)		.022
	N	40	40
VAR00002	Pearson Correlation	.361*	1
	Sig. (2-tailed)	.022	
	N	40	40

*. Correlation is significant at the 0.05 level (2-tailed).

```
CORRELATIONS
  /VARIABLES=VAR00001 VAR00002
  /PRINT=TWOTAIL NOSIG
  /MISSING=PAIRWISE.
```

Correlations

Notes

Output Created		13-MAR-2018 06:54:49
Comments		
Input	Active Dataset	0
	Filter	<none>
	Weight	<none>
	Split File	<none>
	N of Rows in Working Data File	40
Missing Value Handling	Definition of Missing	User-defined missing values are treated as missing.
	Cases Used	Statistics for each pair of variables are based on all the cases with valid data for that pair.
Syntax		CORRELATIONS /VARIABLES=VAR00001 VAR00002 /PRINT=TWOTAIL NOSIG...
Resources	Processor Time	00:00:00.02
	Elapsed Time	00:00:00.01

[0]

Correlations

		VAR00001	VAR00002
VAR00001	Pearson Correlation	1	.323*
	Sig. (2-tailed)		.042
	N	40	40
VAR00002	Pearson Correlation	.323*	1
	Sig. (2-tailed)	.042	
	N	40	40

*. Correlation is significant at the 0.05 level (2-tailed).

```
CORRELATIONS
  /VARIABLES=VAR00001 VAR00002
  /PRINT=TWOTAIL NOSIG
  /MISSING=PAIRWISE.
```


```
CORRELATIONS
  /VARIABLES=VAR00001 VAR00002
  /PRINT=TWOTAIL NOSIG
  /MISSING=PAIRWISE.
```


Correlations

Notes

Output Created		13-MAR-2018 06:58:39
Comments		
Input	Active Dataset	0
	Filter	<none>
	Weight	<none>
	Split File	<none>
	N of Rows in Working Data File	40
Missing Value Handling	Definition of Missing	User-defined missing values are treated as missing.
	Cases Used	Statistics for each pair of variables are based on all the cases with valid data for that pair.
Syntax		CORRELATIONS /VARIABLES=VAR00001 VAR00002 /PRINT=TWOTAIL NOSIG...
Resources	Processor Time	00:00:00.02
	Elapsed Time	00:00:00.01

[0]

Correlations

		VAR00001	VAR00002
VAR00001	Pearson Correlation	1	.371*
	Sig. (2-tailed)		.018
	N	40	40
VAR00002	Pearson Correlation	.371*	1
	Sig. (2-tailed)	.018	
	N	40	40

*. Correlation is significant at the 0.05 level (2-tailed).

```
CORRELATIONS
  /VARIABLES=VAR00001 VAR00002
  /PRINT=TWOTAIL NOSIG
  /MISSING=PAIRWISE.
```

Correlations

Notes

Output Created		13-MAR-2018 06:59:03
Comments		
Input	Active Dataset	0
	Filter	<none>
	Weight	<none>
	Split File	<none>
	N of Rows in Working Data File	40
Missing Value Handling	Definition of Missing	User-defined missing values are treated as missing.
	Cases Used	Statistics for each pair of variables are based on all the cases with valid data for that pair.
Syntax		CORRELATIONS /VARIABLES=VAR00001 VAR00002 /PRINT=TWOTAIL NOSIG...
Resources	Processor Time	00:00:00.00
	Elapsed Time	00:00:00.01

[0]

Correlations

		VAR00001	VAR00002
VAR00001	Pearson Correlation	1	.359*
	Sig. (2-tailed)		.023
	N	40	40
VAR00002	Pearson Correlation	.359*	1
	Sig. (2-tailed)	.023	
	N	40	40

*. Correlation is significant at the 0.05 level (2-tailed).

```
CORRELATIONS
  /VARIABLES=VAR00001 VAR00002
  /PRINT=TWOTAIL NOSIG
  /MISSING=PAIRWISE.
```

Correlations

Notes

Output Created		13-MAR-2018 06:59:14
Comments		
Input	Active Dataset	0
	Filter	<none>
	Weight	<none>
	Split File	<none>
	N of Rows in Working Data File	40
Missing Value Handling	Definition of Missing	User-defined missing values are treated as missing.
	Cases Used	Statistics for each pair of variables are based on all the cases with valid data for that pair.
Syntax		CORRELATIONS /VARIABLES=VAR00001 VAR00002 /PRINT=TWOTAIL NOSIG...
Resources	Processor Time	00:00:00.00
	Elapsed Time	00:00:00.01

[0]

Correlations

		VAR00001	VAR00002
VAR00001	Pearson Correlation	1	.369*
	Sig. (2-tailed)		.019
	N	40	40
VAR00002	Pearson Correlation	.369*	1
	Sig. (2-tailed)	.019	
	N	40	40

*. Correlation is significant at the 0.05 level (2-tailed).

```
CORRELATIONS
  /VARIABLES=VAR00001 VAR00002
  /PRINT=TWOTAIL NOSIG
  /MISSING=PAIRWISE.
```

Correlations

Notes

Output Created		13-MAR-2018 06:59:37
Comments		
Input	Active Dataset	0
	Filter	<none>
	Weight	<none>
	Split File	<none>
	N of Rows in Working Data File	40
Missing Value Handling	Definition of Missing	User-defined missing values are treated as missing.
	Cases Used	Statistics for each pair of variables are based on all the cases with valid data for that pair.
Syntax		CORRELATIONS /VARIABLES=VAR00001 VAR00002 /PRINT=TWOTAIL NOSIG...
Resources	Processor Time	00:00:00.02
	Elapsed Time	00:00:00.01

[0]

Correlations

		VAR00001	VAR00002
VAR00001	Pearson Correlation	1	.330*
	Sig. (2-tailed)		.037
	N	40	40
VAR00002	Pearson Correlation	.330*	1
	Sig. (2-tailed)	.037	
	N	40	40

*. Correlation is significant at the 0.05 level (2-tailed).

```
CORRELATIONS
  /VARIABLES=VAR00001 VAR00002
  /PRINT=TWOTAIL NOSIG
  /MISSING=PAIRWISE.
```

Correlations

Notes

Output Created		13-MAR-2018 06:59:46
Comments		
Input	Active Dataset	0
	Filter	<none>
	Weight	<none>
	Split File	<none>
	N of Rows in Working Data File	40
Missing Value Handling	Definition of Missing	User-defined missing values are treated as missing.
	Cases Used	Statistics for each pair of variables are based on all the cases with valid data for that pair.
Syntax		CORRELATIONS /VARIABLES=VAR00001 VAR00002 /PRINT=TWOTAIL NOSIG...
Resources	Processor Time	00:00:00.03
	Elapsed Time	00:00:00.01

[0]

Correlations

		VAR00001	VAR00002
VAR00001	Pearson Correlation	1	.366*
	Sig. (2-tailed)		.020
	N	40	40
VAR00002	Pearson Correlation	.366*	1
	Sig. (2-tailed)	.020	
	N	40	40

*. Correlation is significant at the 0.05 level (2-tailed).

```
CORRELATIONS
  /VARIABLES=VAR00001 VAR00002
  /PRINT=TWOTAIL NOSIG
  /MISSING=PAIRWISE.
```

Correlations

Notes

Output Created		13-MAR-2018 07:00:03
Comments		
Input	Active Dataset	0
	Filter	<none>
	Weight	<none>
	Split File	<none>
	N of Rows in Working Data File	40
Missing Value Handling	Definition of Missing	User-defined missing values are treated as missing.
	Cases Used	Statistics for each pair of variables are based on all the cases with valid data for that pair.
Syntax		CORRELATIONS /VARIABLES=VAR00001 VAR00002 /PRINT=TWOTAIL NOSIG...
Resources	Processor Time	00:00:00.00
	Elapsed Time	00:00:00.01

[0]

Correlations

		VAR00001	VAR00002
VAR00001	Pearson Correlation	1	.368*
	Sig. (2-tailed)		.019
	N	40	40
VAR00002	Pearson Correlation	.368*	1
	Sig. (2-tailed)	.019	
	N	40	40

*. Correlation is significant at the 0.05 level (2-tailed).
