# Supplementary material for: Novel Digital Features Discriminate Between Drought Resistant and Drought Sensitive Rice Under Controlled and Field Conditions
Source: Front Plant Sci. 2018 Apr 17;9:492. doi: 10.3389/fpls.2018.00492 (PMC5913589; doi:10.3389/fpls.2018.00492)
Supplement: Supplementary Presentation 8 — The details about the maize experiment and results. [file Presentation8.PDF]

To evaluate the ability of the drought-related image features to report drought response in maize, 347 accessions were planted singly in pots. Each accession was represented by two individuals, one of which was well-watered throughout the experiment (Control Group) and the other one subjected to drought stress (Drought Group). Both groups were watered for the first 23 days after planting. After day 24, no further water was supplied to the drought group. From day 26 to day day 52, RGB images were subsequently taken at 3 day intervals by RAP (Yang et al., 2014).

PAR values were calculated over the 9 time points for all accessions grown under two water treatments (control and drought). In general, the PAR value of control group decreases over the 9 time points (Supplementary Figure 1B), because the maize plant grows larger normally and the growth rate of plant perimeter is less than the growth rate of projected plant area. Compared with control group, the PAR value of drought group increased from the D5 time point, which meant the leaf-rolling of most of maize accessions start (Supplementary Figure 1A). An example (accession *05W002*) is shown in Supplementary Figure 1C.

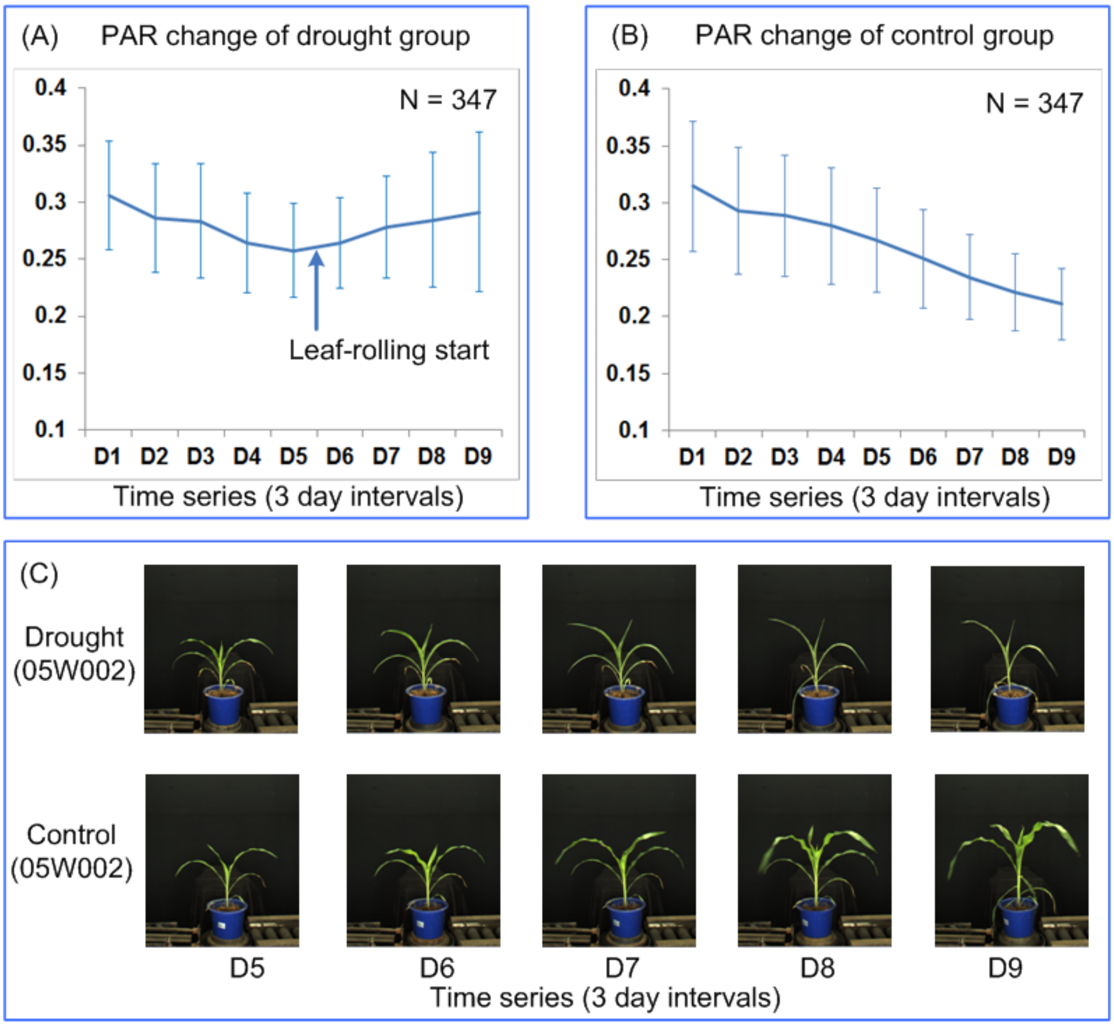

Supplementary Figure 1 **Quantification of leaf-rolling for 347 maize accessions at 3 day intervals.** dynamics of PAR for (A) drought group and (B) control group. The markers and the bars in each line represent the mean value and standard deviation across the accessions, respectively. (C) growth images for a maize accession from D5 to D9.
